# Orthotopic and heterotopic murine models of pancreatic cancer and their different responses to FOLFIRINOX chemotherapy

**DOI:** 10.1242/dmm.034793

**Published:** 2018-07-30

**Authors:** Derek J. Erstad, Mozhdeh Sojoodi, Martin S. Taylor, Sarani Ghoshal, Allen A. Razavi, Katherine A. Graham-O'Regan, Nabeel Bardeesy, Cristina R. Ferrone, Michael Lanuti, Peter Caravan, Kenneth K. Tanabe, Bryan C. Fuchs

**Affiliations:** 1 Department of Surgery, Massachusetts General Hospital, Harvard Medical School, Boston, MA 02114, United States; 2 Department of Pathology, Massachusetts General Hospital, Harvard Medical School, Boston, MA 02114, United States; 3 Martinos Center for Biomedical Imaging, Massachusetts General Hospital, Harvard Medical School, Charlestown, MA 02129, United States; 4 Institute for Innovation in Imaging, Massachusetts General Hospital, Boston, MA 02114, United States

**Keywords:** PDAC animal model, Surgical technique, Chemoresistance, Orthotopic, Heterotopic

## Abstract

Syngeneic, immunocompetent allograft tumor models recapitulate important aspects of the tumor microenvironment and have short tumor latency with predictable growth kinetics, making them useful for trialing novel therapeutics. Here, we describe surgical techniques for orthotopic and heterotopic pancreatic ductal adenocarcinoma (PDAC) tumor implantation and characterize phenotypes based on implantation site.

Mice (*n*=8 per group) were implanted with 10^4^ cells in the pancreas or flank. Hy15549 and Han4.13 cell lines were derived from primary murine PDAC (Ptf1-Cre; LSL-KRAS-G12D; Trp53 Lox/+) on C57BL/6 and FVB strains, respectively. Single-cell suspension and solid tumor implants were compared. Tumors were treated with two intravenous doses of FOLFIRINOX and responses evaluated.

All mice developed pancreatic tumors within 7 days. Orthotopic tumors grew faster and larger than heterotopic tumors. By 3 weeks, orthotopic mice began losing weight, and showed declines in body condition requiring euthanasia starting at 4 weeks. Single-cell injection into the pancreas had near 100% engraftment, but solid tumor implant engraftment was ∼50% and was associated with growth restriction. Orthotopic tumors were significantly more responsive to intravenous FOLFIRINOX compared with heterotopic tumors, with greater reductions in size and increased apoptosis. Heterotopic tumors were more desmoplastic and hypovascular. However, drug uptake into tumor tissue was equivalent regardless of tumor location or degree of fibrosis, indicating that microenvironment differences between heterotopic and orthotopic tumors influenced response to therapy.

Our results show that orthotopic and heterotopic allograft locations confer unique microenvironments that influence growth kinetics, desmoplastic response and angiogenesis. Tumor location influences chemosensitivity to FOLFIRINOX and should inform future preclinical trials.

This article has an associated First Person interview with the first author of the paper.

## Abstract (precis)

**Summary:** Orthotopic and heterotopic allograft locations confer unique microenvironments that influence growth kinetics, desmoplasia and angiogenesis. These differences translate to differential sensitivities to FOLFIRINOX and should inform future preclinical study design.

## INTRODUCTION

Despite advances, pancreatic ductal adenocarcinoma (PDAC) continues to have a poor 5-year survival of 5-7% (SEER Stat Fact Sheets, 2014; Ryan et al., 2014). Surgical resection offers the only potential for cure; however, more than 80% of patients present with either locally advanced or metastatic disease. A number of factors contribute to the aggressive nature of pancreatic cancer, including a high rate of *KRAS*-activating mutations (>90%) (Andre et al., 2002), cellular metabolism reprogramming (Son et al., 2013) and evasion of tumor immunity (Feig et al., 2012). In addition, PDAC is notable for an intense fibrotic reaction associated with the tumor, known as the desmoplastic reaction (Armstrong et al., 2004; Linder et al., 2001; Mollenhauer et al., 1987). In recent years, a plethora of new potential pharmacologic strategies have been developed for PDAC, including mutation-targeted therapy, antistromal agents, immunotherapy and optimized cytotoxic regimens, e.g. FOLFIRINOX (Lim et al., 2012; Heinemann et al., 2013). Although individually these agents can provide modest benefit, a targeted multipathway strategy might hold more promise. Therefore, the availability of preclinical animal models that accurately capture the different pathogenic aspects of the human disease is crucial to move the field forward.

Multiple murine models for pancreatic cancer have been developed. An important distinction is the presence of a functioning immune system. The xenograft model is commonly used, in which immunodeficient mice are transplanted with patient-derived (PDX) tumors or established human cells lines, e.g. BxPC3, Panc-1, MiaPaca-2. A proposed advantage is that human tissue/cells model the disease more accurately. Tumor latency is also short, and predictable growth kinetics allow for reliable measurements of a drug’s response. However, recent evidence suggests that primary tumor xenografts undergo significant genetic changes that diverge from the evolutionary course observed in human disease, calling into question the degree of translatability (Ben-David et al., 2017; Rubio-Viqueira et al., 2006). In addition, although immunotherapy response rates in human PDAC have thus far been poor, there is strong evidence to indicate that immune infiltrates play an important role in shaping the tumor microenvironment and course of disease (Ozdemir et al., 2015). Finally, stromal cells and carcinoma cells are from different species in xenografts, which might influence cell-cell interactions. Thus, immunodeficient models are limited by an incomplete tumor microenvironment.

Another common approach is the use of genetically engineered murine models (GEMMs). Hingorani et al. developed a pancreas-specific GEMM with oncogenic activation in *Kras* (G12D mutation) and simultaneous inactivation of the *Trp53* (R172H) tumor suppressor (Hingorani et al., 2005). This double-hit model reliably produces *de novo* PDAC that mimics the human disease, although it is limited by variable time to development of invasive disease (6-50 weeks). Other groups developed similar models with mutations in *Ink4*/*Arf* (also known as *Cdkn2a*), *Muc1*, *Mist1* (also known as *Bhlha15*), *Smad4* and *Tgfbr2* tumor suppressors (Aguirre et al., 2003; Tinder et al., 2008; Tuveson et al., 2006; Kojima et al., 2007; Ijichi et al., 2006). GEMMs are useful for studying the biology underlying tumorigenesis, local invasion, metastases and immune response, as well as the pathogenic influence of specific gene mutations. However, tumor latency limits their utility for the design and assessment of drug treatment responses.

An alternative approach is the use of tumor allografts. Multiple syngeneic cell lines have been cultured from primary and metastatic PDAC GEMM tumors, which can be subsequently used for controlled surgical implantation, either orthotopically or heterotopically (Tseng et al., 2010). There are several advantages to this approach. Similar to xenografts, tumors have a short latency and reliable growth kinetics. Depending on the cell line, tumors typically maintain a glandular histologic appearance and recapitulate a robust desmoplastic response. Finally, unlike xenograft models, the immune system remains intact and stromal/carcinoma cells are from the same species. Recent advances in our understanding of PDAC pathophysiology have brought the tumor microenvironment to the forefront as a potential therapeutic target, and the allograft model is well suited for this line of investigation.

The quality of a surgical tumor model largely depends on the operative technique and the ability to reliably establish localized disease with predictable growth kinetics. Multiple research teams have published their experiences with surgical models of pancreatic cancer, although details regarding technical pitfalls and procedural insights are lacking. The aim of this paper is to describe the results of different syngeneic, immunocompetent allograft models. First, we provide a detailed analysis of operative techniques for tumor implantation. Second, we describe phenotypic and biological differences in tumors based on implantation location (orthotopic versus heterotopic) and substrate (single-cell suspension versus solid tumor graft), including desmoplastic response, vascularity, immune infiltration and chemosensitivity. Characterization of these differences should serve as a guide for specific applications of the allograft model.

## RESULTS

### Surgical technique to minimize leakage-related carcinomatosis and support reliable tumor growth

**Fig. 1.**
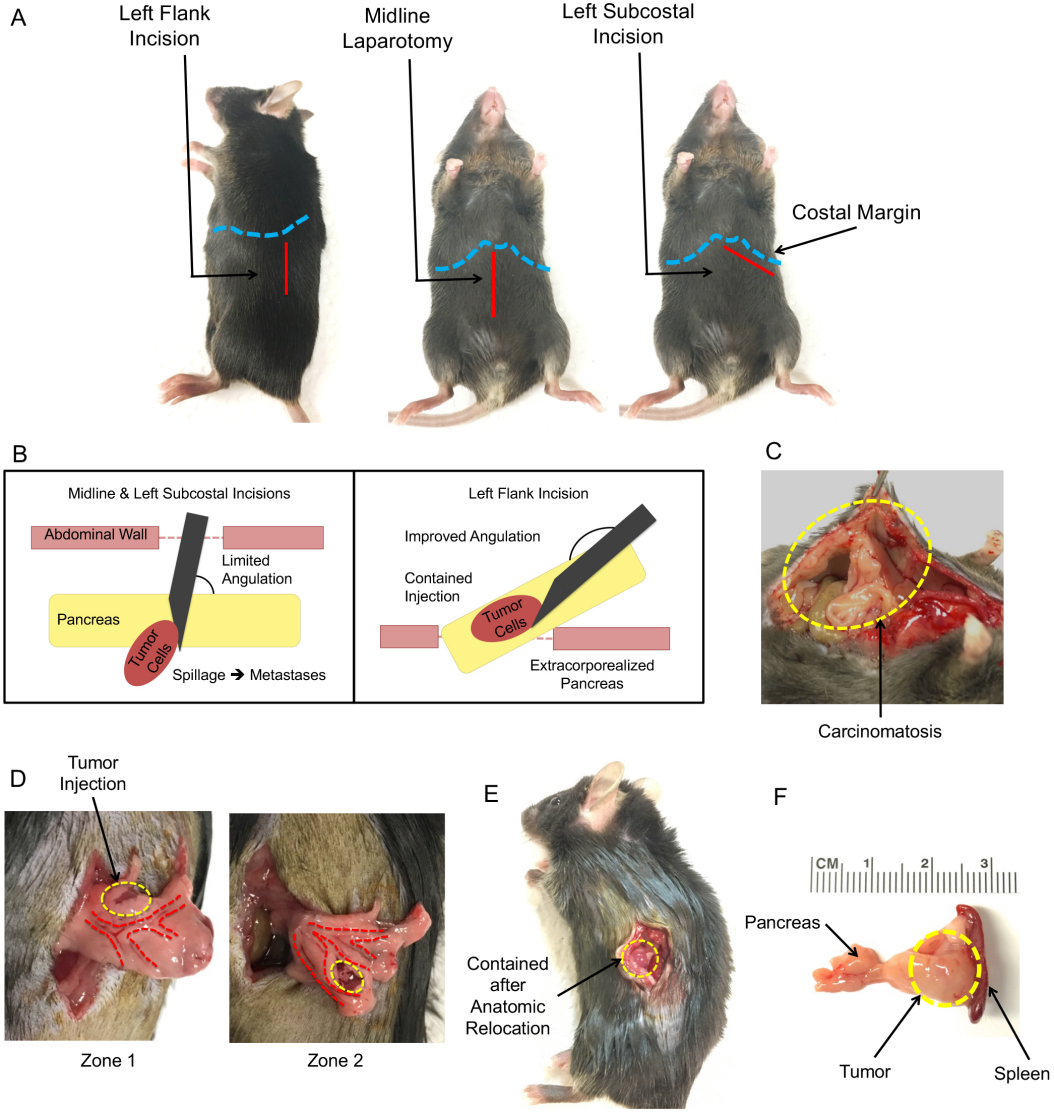
**Surgical technique for orthotopic PDAC tumor injection.** (A) Three surgical incisions (solid red lines) provide exposure to the murine pancreas: left flank incision, midline laparotomy and left subcostal incision. The costal margins are indicated by dashed blue lines. (B) Near-perpendicular needle penetration into the pancreas with midline and left subcostal incisions increases the likelihood of ‘through-and-through’ injuries and spillage of tumor contents. Left-flank incision allows for extracorporealization of the pancreas, providing a superior angle for needle penetration and tumor injection. (C) Carcinomatosis secondary to spillage during injection; the tumor lines the peritoneum and is adhered to the anterior abdominal wall (dashed yellow line). (D) There are two regions (dashed yellow lines) in the body and tail of the pancreas most suitable for injection of malignant cells. Zone 1 is located in the body of the pancreas, superior to large vessels (dashed red lines). Zone 2 is located in the tail of the pancreas, inferior to the large vessels. (E) Representative image of a contained tumor injection (dashed yellow line) after replacement of the spleen and pancreas to their normal anatomic positions. (F) Representative image of orthotopic pancreatic tumor (dashed yellow line) explanted with the spleen and pancreas 2 weeks after injection of syngeneic Hy15549 cells.

Multiple surgical approaches have been proposed for PDAC orthotopic tumor implantation, although detailed evaluations of the strengths and weaknesses of different techniques are lacking. In our experience, there are three main surgical incisions that can be performed: midline laparotomy, left subcostal and left flank (Fig. 1A). All three provide excellent exposure of the murine pancreas and surrounding organs. However, the two anterior approaches (midline and left subcostal) set the operator up for an intracorporeal tumor injection, in which the surrounding intra-abdominal organs and abdominal wall limit the angle of approach with the syringe during tumor injection (Fig. 1B). Consequently, the bevel of the needle penetrates the pancreatic capsule typically at an angle of 45-90°. The pancreas is a small organ of only several millimeters thickness in the anterior-to-posterior direction. Therefore, as the angle of approach nears perpendicularity, the likelihood of driving the bevel of the needle ‘through-and-through’ increases, particularly because the operator is blind to the tip of needle with this approach. An erroneous injection of tumor cells in the retroperitoneal space (posterior to the pancreas) not only defeats the purpose of an orthotopic model, but is likely to result in extravasation of contents into the peritoneal cavity, resulting in carcinomatosis (Fig. 1C).

The volume of injection also affects which angle needs to be utilized. Depending on the number of cells to be injected, which can range from 10^3^ with rapidly growing syngeneic lines to over 10^6^ cells with human xenograft single-cell suspensions, the volume of injection can range from <10 µl to 100 µl. With near-perpendicular penetration into the pancreas, there is little room for driving the bevel of the needle further into the pancreatic parenchyma. This maneuver is very helpful as it lengthens the seal along the needle track, helping to contain the tumor cell contents, which are under pressure after injection. Therefore, the larger the volume of injection, the greater the length of the parenchymal needle track that is necessary in order to counteract the increased pressure of the injected contents, and thereby prevent spillage into the peritoneal cavity. We have observed that leakage is minimal when the needle track is ∼4-5 mm, regardless of volume injected. Arguably, the most common technical error with establishing an orthotopic PDAC model is recurrent spillage of contents and development of carcinomatosis, typically due to a lack of optimization regarding needle penetration angle and injection volume.

We observed that a left-flank incision coupled with gentle pancreatic dissection and extracorporealization of the body and tail of the pancreas and the spleen provides the most reliable method for contained injections of single-cell tumor suspensions (Fig. 1D). With this approach, a 1 cm incision is made in a caudal to rostral direction, ∼5-7 mm anterior to the spine. The rostral edge of the incision should stop just below the costal margin (dashed blue lines, Fig. 1A). Similar to a subcostal incision, there are two layers of tissue to penetrate, including the skin and fat superficially, with abdominal musculature underneath. Blunt dissection of the skin away from the underlying muscle fascia allows for larger muscle flaps to facilitate closure. Unlike a midline laparotomy, there is not a natural fascial plane for dissection. Muscle fibers are cut, which can result in mild bleeding that can be stopped with gentle pressure from a cotton-tipped swab. By placing outward tension on the anterior flap of the incision, the intra-abdominal contents are well visualized, including the pancreas, which is located anterior and inferior to the body of the spleen from this approach.

To mobilize the pancreas, fine-tipped forceps should be used to gently grab the visible edge of the pancreas, which should be pulled out of the incision, bringing the spleen with it. During this dissection of the pancreas, the spleen should never be directly handled with the forceps, which will cause splenic injury and bleeding. This maneuver exposes the connecting ligaments of the murine spleen, which compared with that of humans, is more loosely contained. On the superomedial aspect of the spleen is the gastrosplenic ligament, which appears as a thin film between the greater curve of the stomach and tip of the spleen. This should be cut with scissors, freeing the spleen and pancreas for extracorporealization. The spleen should be laid flat on the posterior incision flap with the pancreas splayed on top (Fig. 1D). With extracorporealization, the operative field should have a wide sterile margin that includes the entire posterior surface of the mouse. The pancreas has a tendency to bunch and fold on itself, which can lead to false passages during injection, resulting in spillage. Therefore, it is paramount to carefully lay out the folds of pancreatic tissue into a single plane. It should be noted that extracorporealization is possible with a midline laparotomy in addition to a left-flank incision. However, in our experience, pancreatic and splenic mobilization is more easily performed via the flank approach.

Once the pancreas has been adequately exposed, the injection path of the needle should be planned. In addition to false passages, inadequate penetration and spillage of contents around the needle track, lacerating a major vein or artery in the pancreas is another pitfall that can result in a large hematoma, affecting tumor growth. The vasculature of the murine pancreas typically presents as shown by the dashed red lines in Fig. 1D. In order to avoid vascular injury, there are two common landing zones (dashed yellow lines, Fig. 1D) for tumor injections that also afford space for a deep needle track to prevent spillage. Zone 1 is located within the body of the pancreas, superior to the major vessels, while Zone 2 is located on the inferior aspect, closer to the tail of the pancreas. Of note, exposure of the head of the pancreas is limited owing to its duodenal attachments, although injection into the head is possible by reversing the direction of injection during the extracorporealized approach.

The needle insertion point and tumor implantation site should be connected by an unobstructed straight line of 4-5 mm in length, thereby providing a more than adequate tunnel to prevent spillage. The direction of the needle track is from anterior-to-posterior on the mouse. For the injection, fine-tipped forceps should be used in the nondominant hand to hold tension on the pancreatic capsule at the site of needle penetration. The angle of insertion should be as close to tangential as possible to prevent a ‘through-and-through’ error, with the bevel of the needle pointed down. We recommend using a 28-gauge insulin syringe for the tumor injection, though a smaller gauge syringe is certainly acceptable as well. We recommend against using a needle larger than 26 gauge, as the risk of leakage from the needle track increases. Once the needle has penetrated the capsule, it should be guided within the parenchyma towards the pre-determined implantation site. If done properly, the needle tip should be visible through the thin layer of covering tissue throughout the course. Tension should be maintained with the forceps in the direction opposite of the path of the needle so as to avoid bunching of the pancreas during insertion. Once the tip of the needle is at the appropriate location, the contents should be gently injected under direct supervision over a duration of 5-10 s. There is often an initial resistance to injection; however, once this is overcome with sufficient pressure, a contained bubble with suspended cells should be observed under the capsule of the pancreas. Friction and catching between the plunger and syringe can result in bursts of excessive pressure that can tear the pancreatic tissue. To avoid this problem, we recommend syringes with hard plastic plungers rather than rubber, as these tend to provide better control during injection. Alternatively, a Hamilton syringe can be used, which also has the advantage of accurate low volume measurements. Once the contents have been fully injected, the needle should be carefully removed along the same trajectory of the entry path while maintaining counter tension with forceps.

Upon completion, there should be a clearly contained fluid bubble within the pancreas (10 µl injection, Fig. 1D). The pancreas and spleen should be gently returned to their normal anatomic positions, which is most easily done by placing lateral tension on the anterior incision flap. The injected tumor, pancreas and spleen shoulder appear as in Fig. 1E at the end of the procedure. Tissue closure is best performed in two layers. We recommend closing the musculature with either 5-0 or 6-0 prolene suture; both running and interrupted fashions are acceptable. For the skin, we recommend surgical clips, as these can be easily and rapidly applied, and they provide a durable closure that is virtually indestructible to mouse tampering. Suture closure of the skin is an acceptable alternative, although these are frequently disrupted by mice, and subsequent wound dehiscence can result in pain, infection and unreliable experimental results. Therefore, suture skin closures need to be closely monitored for integrity for the first postoperative week. An image of the implanted tumor 2 weeks after injection of 10^4^ Hy15549 cells is shown in Fig. 1F.

### Pancreatic tumor growth is predictable and models human disease

Pancreatic tumor uptake is uniformly good in this model with engraftment rates greater than 90%. Tumor growth kinetics are also predictable. We trialed injections with multiple cell counts, including 2×10^6^, 5×10^5^, 1×10^5^ and 1×10^4^ cells (*n*=5 per group), which we evaluated for size on postoperative day (POD) 10. With 2×10^6^ cells, 4/5 mice died from disease prior to the study endpoint. With 5×10^5^ cells, the average tumor diameter was 12.9±4.5 mm on POD10, and mice displayed signs of systemic illness including hunched posture and weight loss. With 1×10^5^ cells, the average tumor diameter was 6.4±1.2 mm, and with 1×10^4^ cells, it was 2.7±0.5 mm. To allow for drug treatments, we determined that 14-21 days was an optimal model duration. Hy15549 and Han4.13 cells exhibited near-identical growth kinetics; therefore, for both cell lines it was determined that 1×10^4^ cells was the optimal inoculum, as this allowed sufficient time for drug treatments without overwhelmingly large tumor masses or death prior to the study endpoint.

**Fig. 2.**
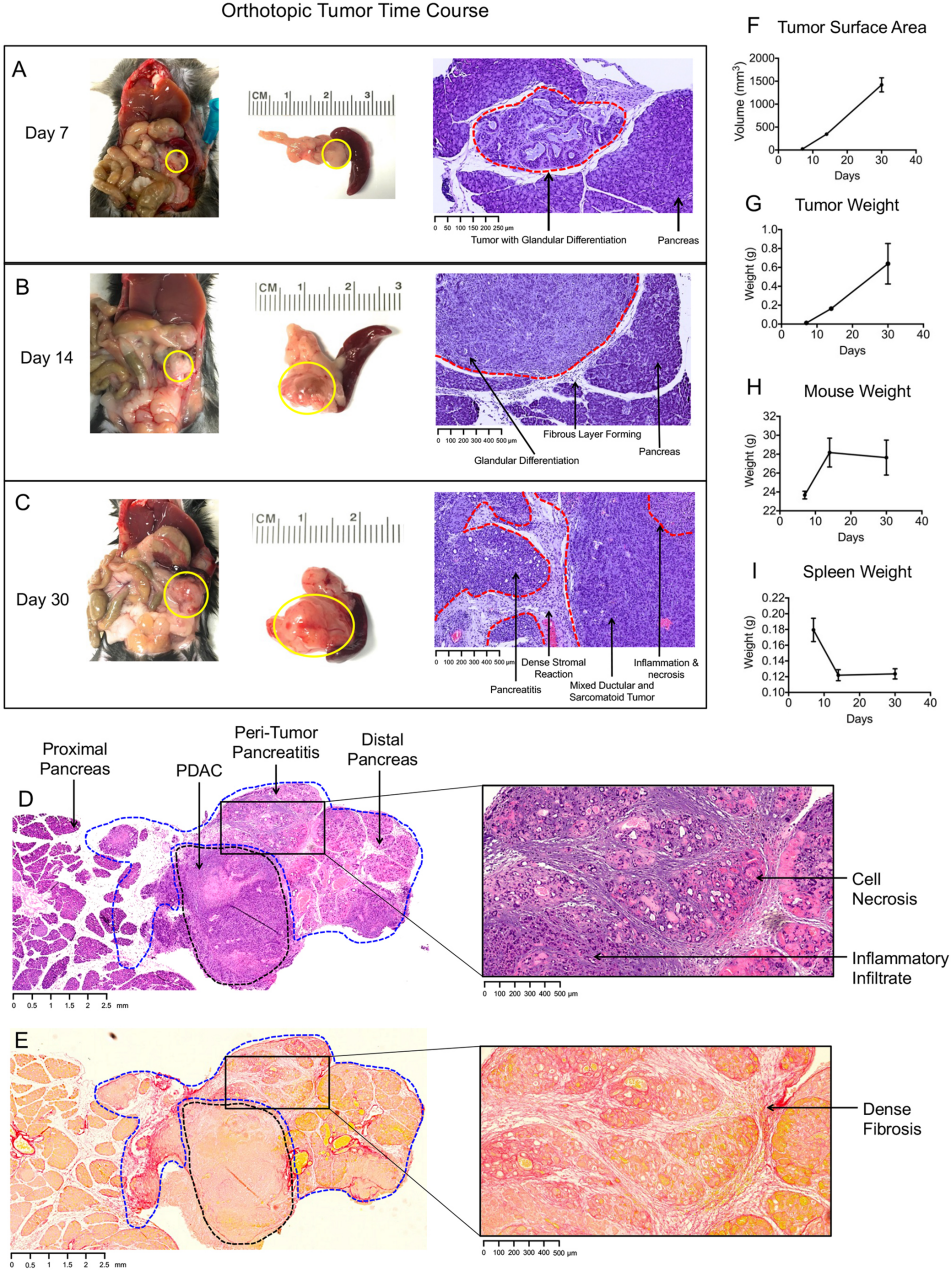
**Orthotopic injection of syngeneic Hy15549 cells recapitulates phenotypic findings of human PDAC.** (A) Gross images of orthotopic PDAC 7 days after injection (*in situ* and explanted; solid yellow lines). H&E-stained slide shows glandular differentiation of cells with residual Matrigel fluid. (B) Tumor at 14 days postinjection; carcinoma cells are more densely clustered, fibrosis is forming around the tumor periphery. (C) Tumor at 30 days postinjection; dense stroma and pancreatitis are now present. (D) Representative H&E of PDAC (dashed black line) and surrounding pancreas (dashed blue line) 2 weeks after injection. Zoomed imaged reveals pancreatitis in the distal pancreas. (E) Sirius Red staining of PDAC and surrounding pancreas. Pancreatitis is associated with a dense fibrotic response. (F) Change in tumor volume over time. (G) Change in tumor weight over time. (H) Change in mouse weight over time. (I) Change in spleen weight over time. *n*=3 for each time point; error bars represent mean±s.d.

Although the implanted tumor cells were preoperatively harvested from a monolayer in plastic cell culture, by POD7, they nonetheless show evidence of polarity, forming abnormal ductular structures as seen in human PDAC (Fig. 2A). Using an inoculum of 10^4^ cells, there is also evidence of stromal deposition occurring by POD7 despite the small tumor size (∼2-3 mm diameter). Although this is not a model of tumorigenesis, it has been previously shown that a desmoplastic reaction is observed in humans as early as the pancreatic intraepithelial neoplasia (PanIN) stage. In our experience, an injection of 10^4^ cells reliably produces a tumor of ∼7-8 mm in diameter by POD14 (Fig. 2B). Interestingly, the tumor architecture and morphology have evolved by this time point, with progressive desmoplasia and increasing glandular complexity.

By POD30, the tumors have grown to 10-15 mm in diameter, and exhibit irregular borders with local invasion into the surrounding retroperitoneum, including the kidney and spleen (Fig. 2C). In addition, 100% of animals had peritoneal and mesenteric metastases, and liver metastases can also be observed at this time point. The tumors have become more vascularized, which is grossly evident by the appearance of vessels coursing through the tumor capsule. Several additional changes have occurred. Owing to the large tumor size, there can be small regions of tumor necrosis with inflammatory infiltrate. The stromal reaction has intensified, with robust fibrotic bands encasing the tumor and surrounding pancreatic tissue. Finally, we observe progressive inflammation, necrosis and fibrosis in the pancreatic tissue surrounding the tumor, consistent with pancreatitis (Fig. 2C). Although pancreatic inflammation and fibrosis are observed surrounding the entire tumor, the majority of pancreatic injury is located distal to the tumor, which is likely due to mechanical compression of the exocrine ducts by the tumor mass. The degree of pancreatitis correlates with tumor size, with larger tumors associated with a more severe pancreatitis response (Fig. 2D,E**)**. Within the inflamed pancreatitis tissue, ductal metaplasia is frequently observed, evidenced by increased cytokeratin-positive immunohistochemical (IHC) staining (Fig. S2). Metaplastic foci have small diameters and dense clustering relative to normal pancreatic ductal architecture, and they can be confused for micrometastases from the implanted tumor. However, it should be noted that metaplasia is a separate process secondary to ongoing pancreatic injury.

Depending on the number of cells injected, orthotopic mice begin losing weight by 3 weeks, and show declines in body condition requiring euthanasia starting at 4 weeks. The rate of tumor growth maintains a linear trend throughout the course of the model when measured by either surface area (Fig. 2F) or weight (Fig. 2G). For this model, we use male C57BL/6 mice aged 8-10 weeks at the time of tumor injection. With 10^4^ cells injected, mice continue to grow and gain weight until approximately POD14 (Fig. 2H). Beyond this time point, however, the animal weights level off and eventually decrease, which is likely a multifactorial process that partly involves reduced appetite. Interestingly, the spleen weight rapidly decreases within the first 2 weeks after tumor implantation (Fig. 2I). On histologic evaluation, there is no evidence of splenic necrosis, and micrometastases are observed. The cause of hyposplenism is not established, although it is likely a result of the local tumor effects on blood flow, specifically compression of the splenic artery and vein.

Taken together, the histologic and gross observations of this model mirror many of the features of human PDAC. Histologically, formation of abnormal ductular structures, progressive and profound stromal response, and surrounding pancreatitis are all features of human PDAC. Similarly, mice in this model lose weight in proportion to tumor burden, with local compression of foregut structures. In the human disease, local tumor effects frequently occur, including duodenal compression and biliary obstruction that contribute to starvation.

### Volume of tumor injection influences subsequent tumor architecture

Tumor cells may be suspended in multiple different solutions for injection, though three commonly used ones include phosphate buffered saline (PBS), cell culture medium such as Dulbecco's modified Eagle medium (DMEM), and Matrigel, which is a complex gelatinous solution composed of scaffolding proteins, glycosaminoglycans and growth factors. Both DMEM and Matrigel provide a nutrient-rich environment to support tumor cell survival and proliferation during the engraftment period. Matrigel, in addition to providing a uniquely supportive nutrient environment, also has the advantage of creating a viscous solution for injection that greatly reduces the risk of leakage from the needle track. For this reason, Matrigel is particularly useful for an orthotopic model. We observed that Matrigel was most effective at leakage reduction when it constituted greater than 50% of the injection solution. Therefore, when resuspending the pellet of cells, one must account for both dilution in Matrigel and the desired volume of injection.

**Fig. 3.**
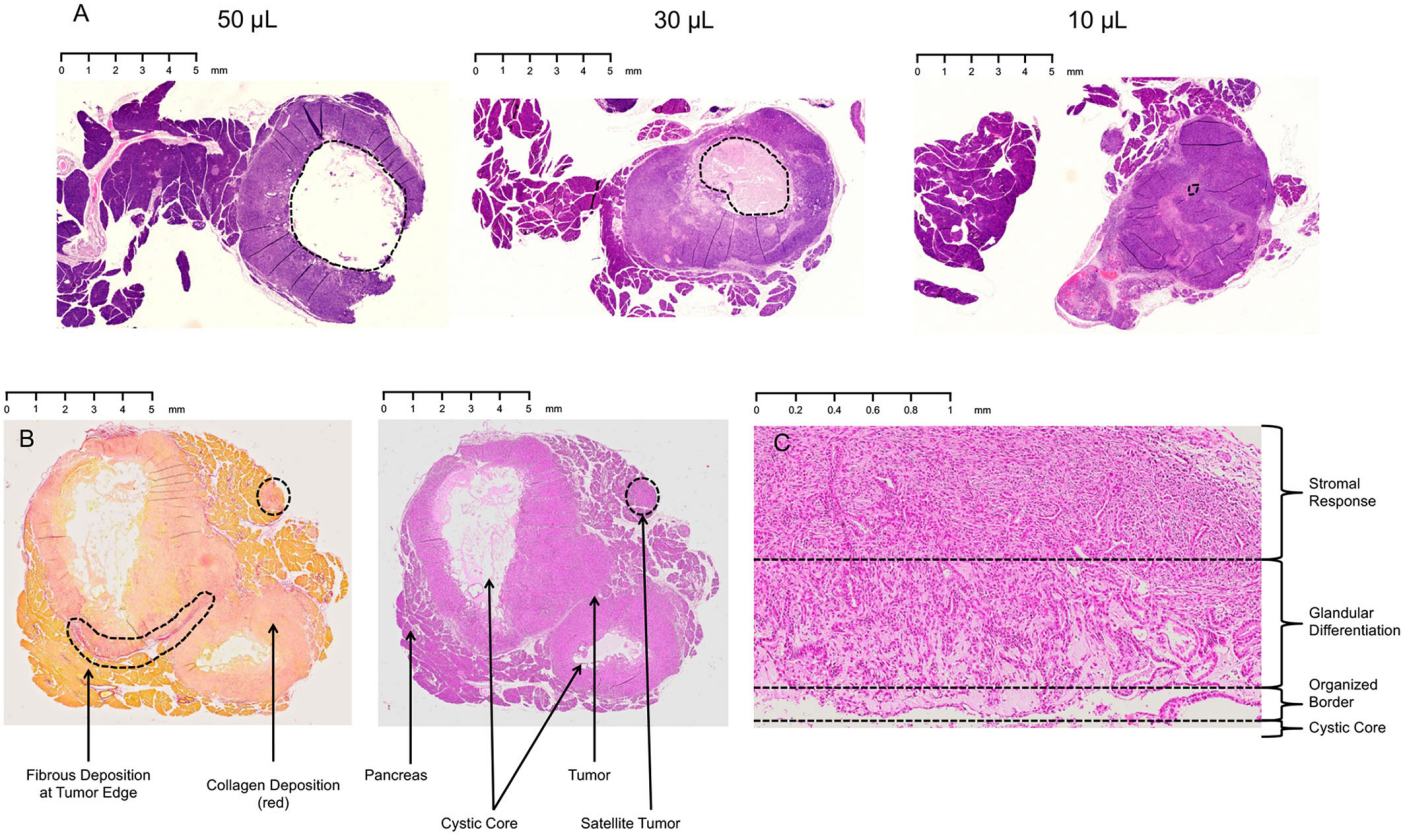
**Injection volume influences tumor architecture and desmoplastic response.** (A) Representative H&E-stained slides of 14-day-old tumors derived from orthotopic injection of 10^4^ Hy15549 cells suspended in 50 µl, 30 µl or 10 µl Matrigel. Increasing Matrigel volume correlates with the size of the cystic center (dashed lines). The effect was abrogated at 10 µl injection volume. (B) Representative Sirius Red- and H&E-stained slides of 14-day-old tumors after 30 µl injection of cells. There is decreased desmoplasia near the cystic center of the tumor. (C) Residual Matrigel is associated with abnormal tumor architecture, including an innermost organized border, followed by glandular differentiation of cells and dense stroma near the periphery.

In this regard, we tested different cellular preparations and injection volumes using Matrigel. One consequence of using a large injection volume (greater than 20 µl) is that tumors form a cystic core, which is proportional in size to the volume injected (Fig. 3A). The initial tumor microenvironment established by Matrigel involves a low-pressure, semiliquid milieu that is rich in collagen, heparin sulfate and growth factors, including tissue growth factor beta (TGF-β) and epidermal growth factor (EGF). In this setting, the innermost tumor cells near the liquid center form an organized border, while the surrounding cells form abnormal ductular architecture (Fig. 3B,C). Interestingly, this inner portion has less stromal response as measured by Sirius Red collagen staining. Further from the center, the tumor becomes more amorphous, and the periphery of the tumor is composed primarily of dense clusters of malignant cells (Fig. 3C). This process was mitigated by reducing the injection volume to ∼10 µl (Fig. 3A). A similar effect occurred with suspensions using DMEM, although not as robust.

### Solid tumor autografts are associated with poor engraftment rates

**Fig. 4.**
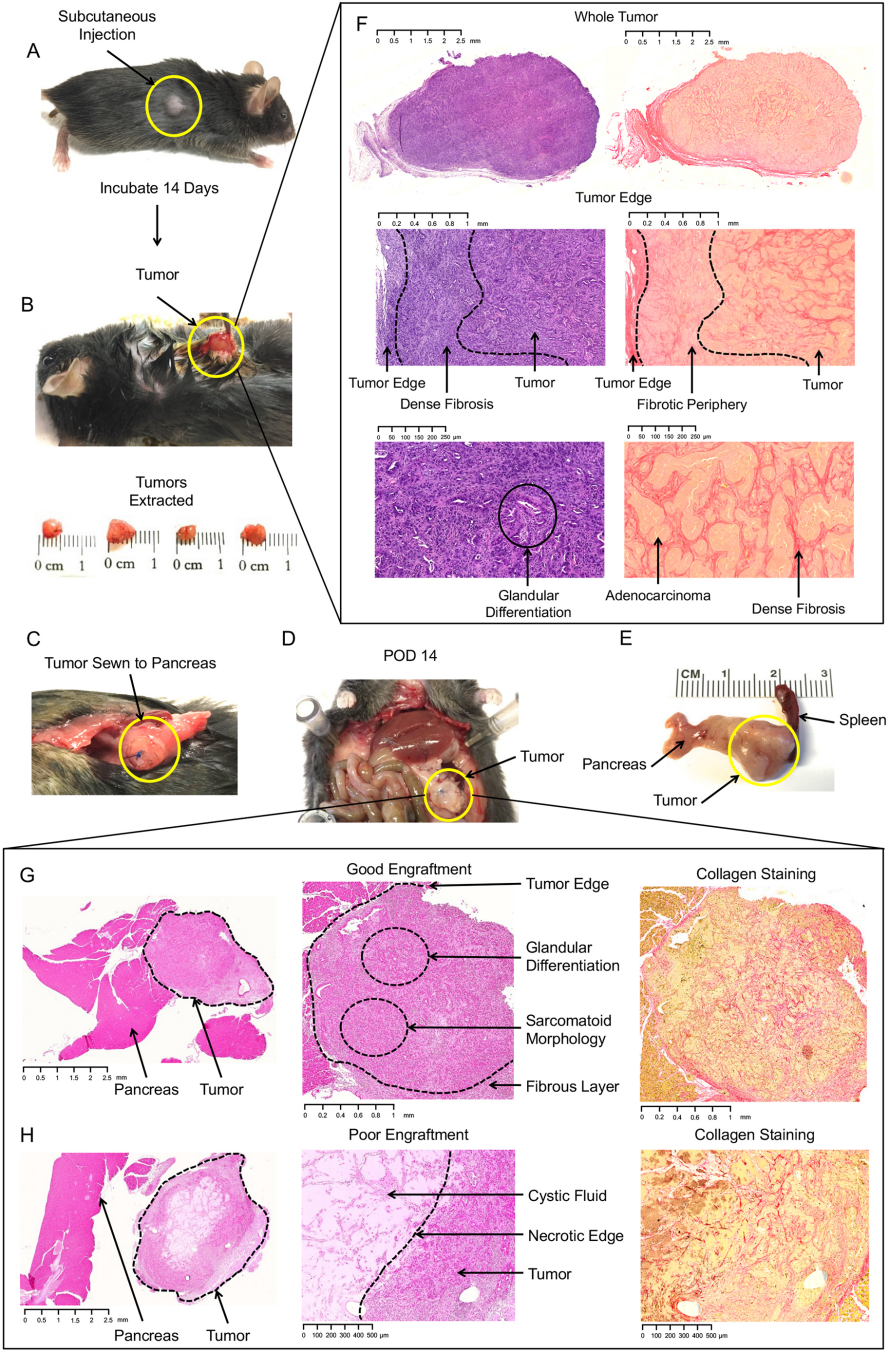
**Surgical technique for orthotopic implantation of solid tumor allograft.** (A) Appearance of wheal (solid yellow line) after subcutaneous injection of tumor liquid suspension. (B) The tumor has formed dense adhesions to the surrounding soft tissue after incubation for 2 weeks. (C) Image of autotransplanted tumor allograft sewn into the body of the pancreas using a 7-0 prolene suture. (D) *In situ* appearance of tumor allograft 2 weeks after implantation. (E) Gross appearance of explanted allograft 2 weeks after implantation. (F) Representative H&E and Sirius Red staining of a heterotopic flank tumor. The flank is associated with a robust fibrotic response that encases and permeates the tumor. (G) Representative H&E and Sirius Red staining of an orthotopically implanted solid tumor allograft. Histologic appearance is similar to lesions in the subcutaneous flank, notable for dense fibrosis. (H) Poor engraftment results in liquefactive necrosis of the allograft.

Another approach towards establishing an orthotopic tumor model is the use of solid tumor autografts initially grown heterotopically. In this method, single-cell tumor suspensions are initially injected into the subcutaneous flank of the animal and allowed to grow for 1-4 weeks, depending on the growth kinetics of the malignant cells (Fig. 4A). Once a mass of ∼4-6 mm has grown, the tumor is surgically explanted from the flank and sewn into the body of the pancreas (Fig. 4B,C). Allograft implantation of solid PDAC tumor tissue directly from a syngeneic GEMM is an alternative approach. In comparison to heterotopic transplants, one advantage of GEMM allografts is that the tumor microenvironment is composed of cells and extracellular matrix (ECM) components naturally found in spontaneously occurring PDAC. However, there is also increased potential for graft rejection as the entire mass is composed of allograft tissue.

There are several technical considerations regarding the surgical technique. First, the heterotopic tumor develops extensive fibrous attachments to the surrounding soft tissue, creating a capsule. Dissection is performed most easily with blunt dissection using fine-tipped scissors. Once the capsular plane is entered, the tumor is easily dissected with minimal blood loss. The skin may be closed with a single interrupted suture or clip. The tumor should then be prepared for orthotopic implantation into the pancreas. In our experience, we have found better engraftment when the capsular surface is removed on the adherent side of the tumor, thereby maximally exposing malignant and stromal cells for attachment to the pancreatic surface. This can easily be performed by removing a layer 1-2 mm in thickness with a scalpel, which is most easily done under 2× magnification.

We recommend using a thin, monofilament, absorbable suture such as Monocryl to sew in the tumor. A single ‘U’ stitch is performed, in which the suture enters through the tumor, followed by a catch of pancreas tissue, and back out the underside of the tumor mass such that the knot is located on the anterior surface (Fig. 4C). The tightness of the suture is important; if too loose, the tumor is not sufficiently opposed to the pancreatic surface, and if too tight, the anchor to the pancreas can tear or cause ischemic injury. Importantly, the path of the stitch must not contain the main pancreatic duct or a large vessel, as both can lead to pancreatic injury and death, confounding any experimentation on the tumor. To avoid these pitfalls, the body and tail of the pancreas are the safest regions for an anchoring suture, which should contain only 2-3 mm of pancreatic tissue so as to prevent injury. Finally, the course of the needle when entering the pancreas should not be perpendicular, but rather near tangential so as to make the bite as shallow as possible, further reducing the risk of entrapping a large duct or vessel.

Once implanted, the autograft is allowed to grow for several weeks. Compared with single-cell direct injections, autografts are smaller at POD14, suggesting slower growth kinetics (Fig. 4D,E). Heterotopic tumors have a unique histologic pattern notable for an extensive desmoplastic reaction (Fig. 4F). This degree of desmoplasia is retained after engraftment into the pancreas (Fig. 4G). Moreover, regardless of the volume injected into the flank, there is rarely a cystic core generated as is seen with larger injections directly into the pancreas. The relative distribution of abnormal ductular structures is less frequent in heterotopic tumors compared with orthotopic lesions.

Successful engraftment is a major challenge when sewing autografts to the pancreas. When engraftment does not occur, the tumor develops a liquefactive necrosis and fails to grow (Fig. 4H). In our experience, this occurs in up to 50% cases, although other reports have documented failure rates up to 80% (Kim et al., 2009). Using smaller solid tumor fragment sizes might increase the rate of engraftment. In summary, the autograft approach has the limitations of requiring multiple procedures, delayed growth kinetics and poor engraftment compared with the single-cell suspension approach. A key advantage of this model, however, is preclusion from developing leakage of tumor cells and carcinomatosis. Finally, autografted solid tumors are highly desmoplastic and might, therefore, have value for assessing particular aspects of tumor fibrosis.

### Orthotopic versus heterotopic PDAC models: differences in desmoplasia, vascularity and growth kinetics

The heterotopic flank tumor model is frequently used by pancreatic cancer researches for its several advantages. First, the model is simple, requiring only a subcutaneous, or in some cases intramuscular, injection of tumor cells. Second, tumor growth can be noninvasively visualized regularly, providing a quick and reliable means of assessing response to a given therapy. Third, the tumor can be easily biopsied before, during and after therapy for various analyses. Finally, treatments can be administered directly to the mass by injection into the body of the tumor. However, an important limitation to this model is the established tumor microenvironment. Orthotopic tumors more accurately recapitulate the surrounding environment, and display more common patterns of disease progression, including weight loss caused by intra-abdominal complications, as well as metastases to the lung and liver. Moreover, several research teams have previously demonstrated gene expression differences in heterotopic flank versus orthotopic murine tumors derived from the same source, including cell lines and xenografts.

Given these previous observations, we further investigated differences in orthotopic and heterotopic tumors by comparing growth kinetics, desmoplastic response and vascularity using the Hy15549 cell line in immunocompetent mice. C57BL6 males aged 10 weeks were injected with 10^4^ cells in either the left flank or directly into the body of the pancreas. Animals were sacrificed after 2 weeks and tumor tissues were analyzed.

**Fig. 5.**
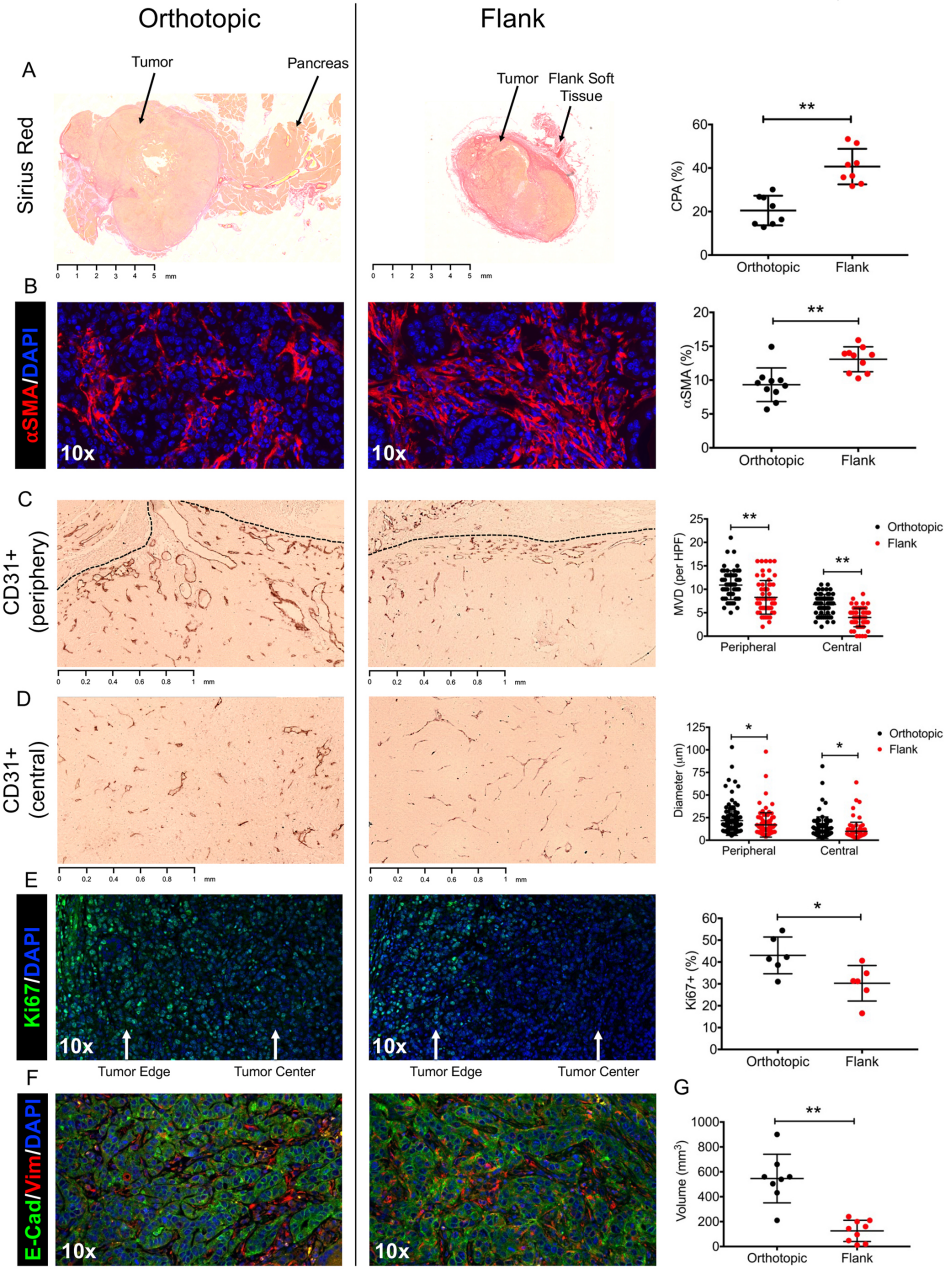
**Phenotypic differences between orthotopic and heterotopic tumors.** (A) Representative Sirius Red staining of orthotopic and heterotopic tumors 2 weeks after injection of 10^4^ Hy15549 cells. Collagen proportional area (CPA) is greater in heterotopic tumors, consistent with increased fibrosis (*n*=8 per experimental group; error bars represent mean±s.d.). (B) Representative immunofluorescent staining for αSMA, which is significantly more abundant in heterotopic tumors. (C) Representative CD31 endothelial staining for vasculature in the periphery or orthotopic and heterotopic tumors. Mean vessel density (MVD) is greater in orthotopic tumors. (D) Representative CD31 endothelial staining for vasculature in the central region of orthotopic and heterotopic tumors. Mean vessel diameter is also significantly greater in orthotopic tumors. (E) Representative immunofluorescent staining for Ki67 (also known as Mki67), which is significantly more abundant in orthotopic tumors. (F) Representative immunofluorescent staining for EMT markers E-cadherin and vimentin; there was no significant difference in staining. (G) Tumor volumes (m^3^) for orthotopic and heterotopic locations. 10× magnification was used for all immunofluorescent images. *P<0.05, **P<0.01.

Heterotopic tumors appeared growth restricted compared with orthotopic tumors as measured by tumor volume (546±66 mm^3^ vs 140±28 mm^3^, *P*<0.0001) (Fig. 5G). In addition, tumor cell proliferation measured by Ki67 immunofluorescent (IF) staining was significantly lower in heterotopic tumors, consistent with the decreased size (percentage of positive cells, 43.1±3.4% vs 30.3±3.3%, *P*=0.023) (Fig. 5E).

Heterotopic tumors were also associated with a more intense desmoplastic reaction compared with orthotopic tumors, evidenced by thicker surrounding fibrous capsules, and more frequent deposition of collagen fibers throughout the tumor parenchyma (Fig. 5A). Accordingly, measurement of the collagen proportional area (CPA) by Sirius Red staining was notable for significant collagen content in heterotopic tumors (40.7±2.9% vs 20.5±2.4%, *P*<0.0001). ECM proteins, including type I collagen, are primarily deposited by activated myofibroblastic cells. In the soft tissue of the flank, this primarily involves dermal fibroblasts, while resident pancreatic stellate cells are the main source of stromal contents in the pancreas. IF staining for alpha smooth muscle actin (αSMA), a marker of myofibroblastic activation, was significantly greater in heterotopic tumors (13.3±0.6% vs 9.3±0.8%, *P*=0.0012), suggesting greater density of activated stromal cells, consistent with the collagen staining (Fig. 5B). Finally, we investigated change in tumor fibrosis over time in the orthotopic model, as one potential concern with a short duration model is limited capacity for stellate cell stimulation, proliferation and desmoplastic activation in the tumor microenvironment. We measured tumor CPA at days 10, 16 and 30 after implantation, and found that fibrosis significantly increased up to POD16, after which time collagen deposition relative to tumor mass appeared to stabilize, as there was no difference in CPA between days 16 and 30 (Fig. S3). These data suggest that ∼2 weeks’ duration is required for establishment of the desmoplastic response in this model system.

Previous reports have shown a relationship between the degree of tumor desmoplasia and hypovascularity. IHC staining of the endothelial marker CD31 (also known as PECAM1) revealed differences in both the mean vessel density and the average vessel diameter between tumor locations (Fig. 5C,D). In general, tumors were found to have a higher vessel density in the periphery compared with central regions, suggesting a spatially heterogeneous distribution of angiogenesis. Vessels in the periphery were also associated with a greater average vessel diameter, which could have a multifactorial etiology, although increased intratumoral pressure likely contributes. When separated by the periphery and central regions of a tumor, both vessel density and diameter were reduced in the heterotopic tumors, suggesting a more hypovascular microenvironment.

We evaluated whether these difference in the tumor microenvironment, particularly the increased desmoplasia and hypovascularity observed in heterotopic tumors, induced epithelial-to-mesenchymal transition (EMT). The Ptf1-Cre; LSL-KRAS (G12D); Trp53 Lox/+ (KPC)-based cell line is epithelial when grown in culture, with high expression of E-cadherin and low expression of vimentin. Although we observed increased vimentin staining after implantation, there did not appear to be a difference in the expression of E-cadherin (Fig. 5F).

Finally, we evaluated whether these results were replicable using a different cell line, Han4.13, which is also derived from a Ptf1-Cre; LSL-KRAS-G12D; p53 Lox/+ genetic background, though is syngeneic with FVB mice. We observed similar phenotypic differences in Han4.13 allografts based on tumor location (Fig. S4). Specifically, orthotopic tumors were larger and less desmoplastic, and pancreatitis was again present surrounding the tumor. However, when grown in culture, the Han4.13 cell line has more mesenchymal features compared with the Hy15549 line, which manifested as less glandular differentiation and more sarcomatoid cell clusters on histologic evaluation of both heterotopic and orthotopic tumors.

### Differential responses to treatment based on tumor location

**Fig. 6.**
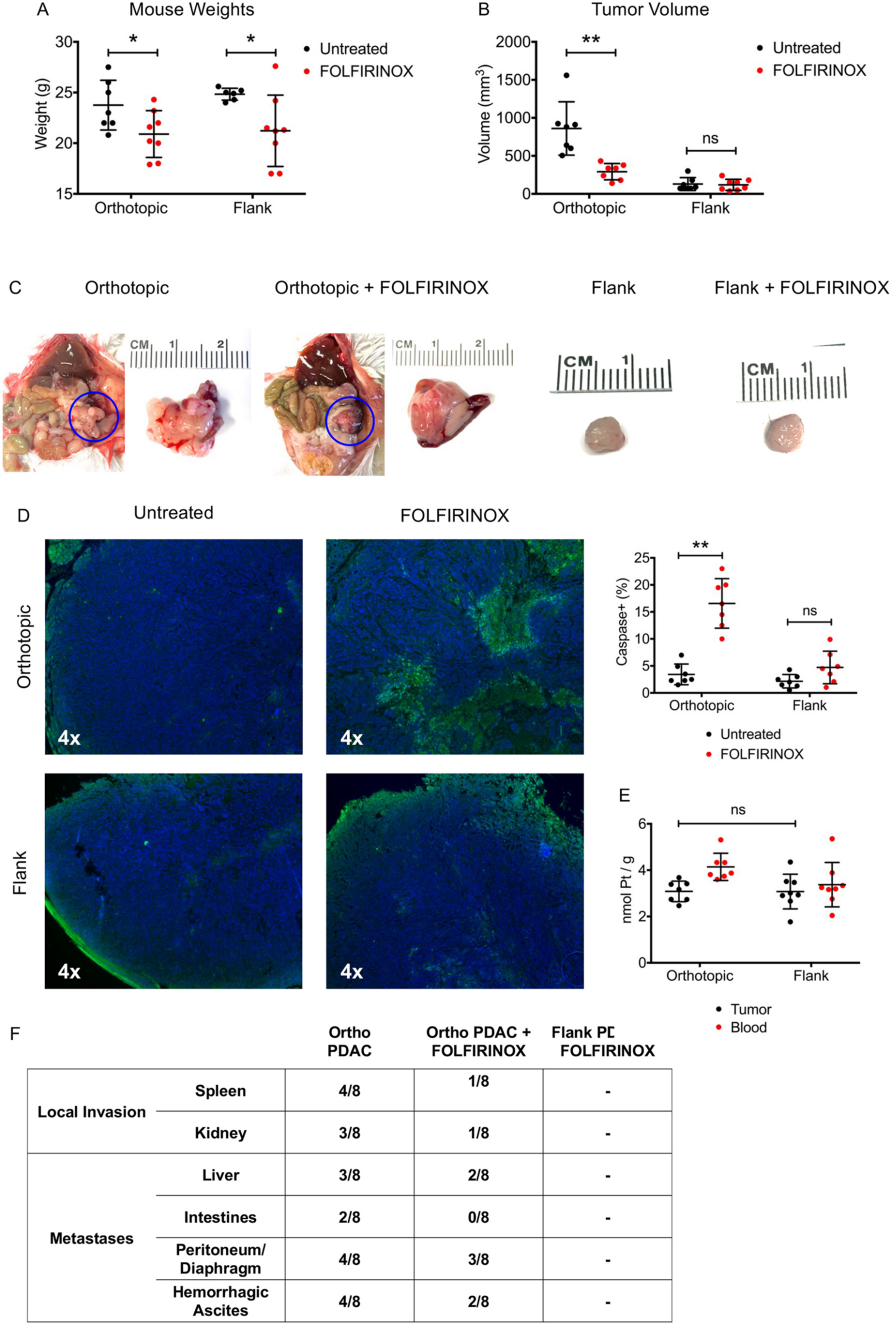
**Tumor location determines response to intravenous FOLFIRINOX chemotherapy.** (A) Mice treated with FOLFIRINOX lost similar amounts of weight regardless of tumor location (*n*=8 per experimental group; error bars represent mean±s.d.). (B) FOLFIRINOX treatment resulted in significant reduction in orthotopic tumor volume, though no difference was observed with heterotopic tumors. (C) Representative gross images (*in situ* and explanted) of orthotopic (solid blue lines) and heterotopic tumors with and without FOLFIRINOX treatment. (D) Representative immunofluorescent staining for cleaved caspase-3; caspase expression is significantly increased in orthotopic but not heterotopic tumors. (E) There was no difference in tumor platinum level 2 h after FOLFIRINOX tail vein injection, suggesting similar levels of drug penetration between orthotopic and heterotopic tumors. (F) FOLFIRINOX treatment was associated with reductions in local tumor invasion and metastatic disease. 4× magnification was used for all immunofluorescent images. *P<0.05, **P<0.01; ns, nonsignificant.

Differences in the tumor microenvironments of orthotopic and heterotopic tumors influenced the response to cytotoxic chemotherapy with FOLFIRINOX. C57BL6 male mice aged 10 weeks were injected with 10^4^ Hy15549 cells in either the flank or directly into the body of the pancreas. FOLFIRINOX was administered by intravenous tail vein injection on POD9 and POD12. Mice were sacrificed on POD14 and the tumors from each group were analyzed. There were no deaths. FOLFIRINOX treatment was associated with similar amounts of weight loss in both tumor groups (Fig. 6A).

FOLFIRINOX treatment was associated with a significant reduction in orthotopic tumor volume compared with untreated controls (860.0±351.1 mm^3^ vs 291.7±107.4 mm^3^, *P*=0.0015) (Fig. 6B). On gross evaluation, FOLFIRINOX-treated orthotopic tumors demonstrated evidence of hemorrhagic necrosis (Fig. 6C). Histologically, orthotopic tumors treated with FOLIRINOX displayed significantly greater amounts of the apoptotic marker, cleaved caspase-3, as measured by the percentage of positive cells (16.6±4.5% vs 3.4±1.9%, *P*<0.0001), consistent with tumor cell death and decreased tumor volume (Fig. 6D). FOLFIRINOX treatment was also associated with reduced local tissue invasion and distant metastases (Fig. 6F).

In contrast, there was a nonsignificant reduction in heterotopic tumor volume after FOLFIRINOX treatment (129.4±84.4 mm^3^ vs 118.5±72.5 mm^3^, *P*=0.79) (Fig. 6B,C). Heterotopic tumors treated with FOLFIRINOX displayed no significant increases in cleaved caspase-3 (4.7±3.0% vs 2.2±1.2%, *P*=0.06) (Fig. 6D), consistent with the overall reduced response to cytotoxic therapy.

Given the increased desmoplastic response and hypovascularity associated with heterotopic tumors, we hypothesized that the decreased response to FOLFIRINOX therapy was a result of decreased drug penetration into the tumor. Therefore, we evaluated platinum concentrations in blood and tumor tissue in heterotopic and orthotopic tumors 2 h after intravenous FOLFIRINOX administration by inductively coupled mass spectrometry (ICP-MS). Platinum metal is a key structural element to the drug oxaliplatin, a component of the FOLIFIRINOX regimen, and is otherwise found at near undetectable levels in rodents and humans. Interestingly, there was no difference in the concentration of platinum in either heterotopic or orthotopic tumors 2 h after injection, suggesting equivalent levels of chemotherapy uptake (orthotopic 3.1±0.4 nmol/g vs 3.1±0.7 nmol/g, *P*=0.97) (Fig. 6E). Given this finding, it appears that microenvironment factors associated with heterotopic tumor injection confer FOLFIRINOX chemoresistance in KPC-derived PDAC cells.

## DISCUSSION

In this study, we tested different surgical approaches for creating syngeneic, immunocompetent PDAC allograft tumor models. For orthotopic tumors, we found that single-cell suspensions provide superior engraftment rates and enhanced growth kinetics compared with solid tumor implants. For solid tumor implants, engraftment rates were near 50%, and other groups have reported rates as low as 20%, which represents a potential problem in terms of wasted time and resources and loss of statistical power among treatment groups (Kim et al., 2009). Regarding operative technique, a left-flank incision with extracorporealization of the pancreas provides optimal exposure for tumor injection compared with a midline laparotomy or left subcostal incision, reducing leakage rates and subsequent carcinomatosis. Finally, injection volume influences subsequent tumor architecture and morphology, and therefore must be taken into consideration. This is particularly true when using Matrigel, which creates a proteinaceous matrix in the core of the tumor. Cells enveloped in Matrigel appeared to retain more glandular architecture, and these regions had less desmoplasia than those in the periphery of the tumor. This effect was abrogated by reducing the volume of injection to ∼10-20 µl.

Heterotopic flank tumors are frequently used by researchers because they can be easily and quickly produced, and they allow for visual monitoring of disease progression and treatment response. However, we found significant biological differences between orthotopic and heterotopic tumors that influenced experimental outcomes. Orthotopic tumors grew faster and larger, and, as expected, they more faithfully produced clinical signs of PDAC, including weight loss and general illness (hunched posture and decreased movement). We also observed surrounding pancreatitis in the orthotopic model, which is likely to influence the local tumor microenvironment, including immune trafficking and the desmoplastic response. Histopathologic evidence of subclinical pancreatitis is also commonly observed in surgically resected specimens of human PDAC (Kohler and Lankisch, 1987; Lin and Feller, 1990). The mechanism for pancreatitis in this context is unknown, though might include mechanical duct obstruction, ischemic injury from vascular compression, and direct activation of pancreatic enzymes by neoplastic cells (Mujica et al., 2000). Histologically, heterotopic tumors had more sarcomatoid-appearing clusters of malignant cells, whereas orthotopic tumors retained more concentrated regions of glandular differentiation. In human PDAC, sarcomatoid appearance is rare, and most cases maintain some degree of glandular differentiation. Finally, heterotopic tumors were more desmoplastic, evidenced by collagen and αSMA staining, which correlated with reduced vascularity by CD31 staining, including both mean vessel density and diameter. This inverse relationship between PDAC desmoplasia and tumor vascularity is corroborated by previous studies. Olive et al. showed that antifibrotic therapy with a smoothened inhibitor reduced tumor stroma and concomitantly increased tumor perfusion and mean vessel density (Olive et al., 2009). Chauhan et al. similarly showed that inhibition of angiotensin II with Losartan resulted in decreased PDAC collagen and hyaluronan content, reducing tumor interstitial pressure, which correlated with increased vascular perfusion (Chauhan et al., 2013).

In support of our histopathologic findings, significant genetic and molecular differences between orthotopic and heterotopic tumor implants have previously been shown. Nakamura et al. published one of the first reports showing that specific organ microenvironments influence gene expression patterns in animal models of PDAC (Nakamura et al., 2007). They performed microarray analyses on three human PDAC lines with different metastatic potential (FG, L3.3, L3.6pl) that were grown *in vitro* or implanted into the subcutaneous flank or pancreas. Gene expression patterns among the different cell lines were similar *in vitro*; however, orthotopic tumors uniquely exhibited increased expression of genes that stimulate cell migration and invasion. More recently, Hoover et al. compared RNA expression profiles of orthotopic and flank tumors, also using a xenograft model with human cell line injections (BxPC3 and FG) and patient-derived PDAC tissue, and found significant differences in the expression of *PEAK1* and *MST1R*, both genes involved in PDAC pathogenesis, based on tumor location (Hoover et al., 2017). Zhan et al. compared metabolic profiles between orthotopic and subcutaneous flank xenografted tumors (Panc-1 and BxPC3 human cell lines). They found differences in purine and lipid processing, as well as ABC transporter function (Zhan et al., 2017). ABC transporters have previously been shown to contribute to PDAC chemoresistance, and were expressed at lower levels in orthotopic tumors (Mohelnikova-Duchonova et al., 2013). Finally, Michaelis et al. compared subcutaneous flank and orthotopic syngeneic KPC allograft tumors for their ability to recapitulate PDAC-associated cachexia (Michaelis et al., 2017). Similar to the gross observations of our current study, orthotopic tumors produced more signs of cachexia compared with flank lesions, including hypothalamic inflammation, loss of brown and white adiposity, increased muscle catabolism and neutrophil-dominant leukocytosis. The importance of systemic sequelae in PDAC models is not well established, although in humans, cachexia is closely associated with disease progression and poor outcome (Bachmann et al., 2008). One advantage of immunocompetent allograft models is the recapitulation of systemic immunologic sequelae, which are lacking with xenograft systems.

Implant location also influenced tumor chemosensitivity, presumably due to microenvironment specific factors, in which heterotopic tumors were less responsive to intravenous FOLFIRINOX. We initially suspected that this was because heterotopic lesions had increased fibrosis and decreased vascularity, and therefore reduced drug penetration. However, drug uptake into tumor tissue was equivalent by mass spectrophotometric analysis, regardless of tumor location or degree of fibrosis, indicating that penetration was not a determinant of chemotherapy response. Although orthotopic tumors were significantly larger at the initiation of chemotherapy, this increased size was unlikely to be a contributor to the increased responsiveness, as we have previously observed similar levels of caspase activation across a range of orthotopic tumor diameters. It has been postulated that PDAC stroma contributes to chemotherapeutic resistance by compressing blood vessels, reducing perfusion and drug delivery to tumor cells. This logic has led to the development of multiple antifibrotic strategies for PDAC (Ozdemir et al., 2015; Stromnes et al., 2014; Thompson et al., 2010). However, subsequent clinical trials with antifibrotic therapy have failed to show a survival benefit (Moore et al., 2003; Bramhall et al., 2002). More recent studies indicate that tumor microenvironment factors might play a crucial role in chemotherapy responsiveness (Koay et al., 2014; Neesse et al., 2013; Ireland et al., 2016). Specifically, it has been shown that activated stellate cells, in addition to the deposition of ECM, also secrete pro-survival and immunosuppressive signaling molecules that might contribute to adenocarcinoma chemoresistance. In humans, stromal activity has been correlated with survival. Erkan et al. developed a pathologic scoring system called the activated stroma index (ASI), which is defined as the ratio of αSMA-stained area to collagen-stained area in the tumor bed (Erkan et al., 2008). They found that high stromal activation, measured by αSMA-positive staining in myofibroblastic cells, relative to low collagen staining, was associated with poor prognosis, whereas high collagen content relative to low αSMA area was associated with good prognosis. Our heterotopic model displayed significantly greater αSMA staining than orthotopic lesions, indicative of greater myofibroblastic activation, which might have contributed to chemoresistance. Moreover, the stroma of heterotopic tumors largely consists of local, dermal fibroblasts which might behave differently to resident stellate cells in the pancreas, with respect to their role in chemoresistance.

Taken together, our findings do not negate the significance of drug penetrance; however, they support the assertion that tumor microenvironment factors play a crucial role in tumor response to cytotoxic therapy. They also highlight the strengths and weaknesses of orthotopic and heterotopic implantation techniques. Human PDAC is associated with extensive desmoplasia, both myofibroblastic stromal activation and fibrosis deposition, and thus heterotopic lesions might better recapitulate the desmoplastic aspects of human PDAC. However, the stroma of heterotopic tumors largely consists of local, dermal fibroblasts. Conversely, orthotopic lesions more accurately portray the microenvironment constituents of PDAC, including resident stellate cells and surrounding inflamed pancreatic tissue. In addition, although the human disease has more extensive desmoplasia than orthotopic murine lesions, considerable cytotoxic activity is still frequently observed after chemotherapy in both contexts.

It should be noted that for both xenografts and allografts, there is concern around the use of cell lines, which have undergone genetic drift after years of selective pressure *in vitro*, and might no longer accurately represent their original tumor source (Knudsen et al., 2017). This is particularly true for well-established human PDAC lines. To counteract this technical drawback, our group performed all experiments between passages 1-8 after cells were acquired from primary murine tumors. Other groups have also developed newer, low-passage human lines from a variety of sources, including ascites (Indolfi et al., 2016). Finally, there is concern that cell culture itself might augment gene expression patterns and subsequent behavior of malignant cells, regardless of baseline mutational faithfulness to the human disease. Therefore, some have proposed organoid-based culturing systems prior to surgical implantation. Further studies are necessary to determine whether this latter approach actually leads to a more clinically accurate animal model.

In conclusion, we believe these insights are valuable as they could impact experimental consistency and outcomes, and there is a paucity of resources in the literature that provide this level of technical characterization. Moreover, although we have focused on characterizing syngeneic, immunocompetent allografts, the operative techniques from this study are relevant to all surgical models of pancreatic cancer, including xenografts, which capture a much larger spread of relevant genetic alterations that have potential for unique biologic insights and, possibly, development of laboratory-based personalized therapeutic regimens.

## MATERIALS AND METHODS

### Animals

All experiments were performed in accordance with the National Institutes of Health (NIH) Guide for the Care of Use of Laboratory Animals and approved by the Massachusetts General Hospital Institutional Animal Care and Use Committee. Eight- to ten-week-old immunocompetent male C57BL/6 mice were obtained from Charles River Laboratories (Wilmington, MA, USA).

### Murine pancreatic cancer cell lines

Hy15549 and Han4.13 cell lines were derived from primary pancreatic tumors from Ptf1-Cre; LSL-KRAS-G12D; p53 Lox/+ mice from the laboratory of Dr Nabeel Bardeesy at Harvard Medical School (Boston, MA, USA). Recovered cells underwent multiple passages to favor the growth of ductal elements over other cellular components and were processed as previously described (Hingorani et al., 2005). Hy15549 and Han4.13 cells are syngeneic with the C57BL/6 and FVB mouse strains, respectively, thus allowing for the creation of immunocompetent tumor models.

### Cell culture

Cells were plated at low density in T75 uncoated plastic flasks and cultured in DMEM (high glucose 4.5 g/l) supplemented with 10% fetal bovine serum and 1% Pen/Strep (100 U/ml penicillin, 100 µg/ml streptomycin). Cells were grown at 37°C in a humidified, 5% CO2 incubator until they reached ∼70% confluence. All experiments were performed between passage 1 and 8.

### Cell preparation for injection

Subconfluent cells were detached with 0.25% trypsin for 2 min at 37°C. The suspended cells were centrifuged at 500 ***g*** for 5 min and resuspended in 1-2 ml DMEM. Cells were counted using a Countess automated cell counter (Invitrogen, Carlsbad, CA, USA). For both Hy15549 and Han4.13 cells, we found the optimal inoculum to be 10^4^ cells in 10 µl Matrigel (10^3^ cells/µl). Given the viscous nature of Matrigel and the tendency for gelatinous components to separate out over time, Matrigel stock solution was split into 100 µl aliquots and stored at −20°C.

### Heterotopic and orthotopic tumor implantation

Heterotopic tumors were created by injecting 10^4^ cells in 10 µl Matrigel under the skin of the left flank. For orthotopic tumors, mice were anesthetized with an intraperitoneal injection of ketamine (100 mg/kg) and xylazine (10 mg/kg). Fur was shaved in the region of the incision using an electric clipper. The surgical field was sterilized with betadine and was allowed to dry for 30 s. We tested two different forms of orthotopic tumor creation: single-cell liquid suspension and subcutaneous tumor transplantation (solid tumor implant engraftment). Details of the surgical procedure are discussed in the Results section of the paper. For the single-cell suspension injections, leakage resulting in peritoneal tumor burden occurred in less than 10% of cases, and these animals were excluded from comparative experimentation with heterotopic tumors. After orthotopic tumor implantation, mice were monitored daily for disease progression by abdominal palpation, as well as for morbidity, evidenced by decreased grooming, hunched posture and reduced movement. For all experiments, *n*=7 mice per treatment group were included unless otherwise noted.

### Tumor growth kinetics

In order to characterize the orthotopic model over time, mice with implanted tumors (*n*=3) were sacrificed at days 7, 14 and 30 to measure tumor volume (mm^3^) and tumor weight (g), and for histopathologic tumor evaluation. Mouse body and spleen weights were also measured at each time point.

### FOLFIRINOX chemotherapy treatment

FOLFIRINOX is a combination drug including Ca^+2^ folinate, oxaliplatin, irinotecan and 5-fluorouracil. Based on previous publications, we trialed the following drug concentrations: Ca^+2^ folinate 100 mg/kg, oxaliplatin 5 mg/kg, irinotecan 50 mg/kg and 5-fluorouracil 50 mg/kg. Healthy C57BL/6 mice, males aged 10 weeks (*n*=5), were given this drug regimen in two doses spaced 3 days apart, which was found to carry significant toxicity resulting in weight loss and animal mortality (Fig. S1). Therefore, the dose was reduced by 50% (Ca^+2^ folinate 50 mg/kg, oxaliplatin 2.5 mg/kg, irinotecan 25 mg/kg and 5-fluorouracil 25 mg/kg), with no deaths from the medication. This lower dose was used for all further experimentation. For studies comparing orthotopic and heterotopic tumor response to FOLFIRINOX, drug was administered on POD10 and POD13. Drug administration was performed by intravenous tail vein injection for 100% bioavailability.

### Histologic analysis

A cardiac terminal blood withdrawal was performed at the time of sacrifice. Pancreas and tumors were extracted from mice at 1, 2, 3 and 4 weeks after orthotopic or heterotopic tumor implantation. Tissues were formalin fixed for 48 h prior to paraffin embedding. Sections were cut at 5 µm thickness. Representative sections were stained with Hematoxylin and Eosin (H&E) and Sirius Red to detect fibrosis. IHC staining for CD31 (1:200; #77699, Cell Signaling Technology, Danvers, MA, USA), alpha smooth muscle actin (αSMA) (1:100; #A5228, Sigma-Aldrich, St Louis, MO, USA) and cleaved caspase-3 (1:200; #9664, Cell Signaling Technology) was performed according to standard protocols. Images were captured with a Nikon Eclipse microscope equipped with an Insight CMOS 5.1 digital camera. Whole slides were scanned using a NanoZoomer-SQ Digital slide scanner (Hamamatsu Photonics K. K, Hamamatsu City, Japan). Sirius Red staining was used to measure CPA for tumor and pancreas samples (Polasek et al., 2012; Fuchs et al., 2013). All image analysis was performed using ImageJ (NIH).

### IHC staining

Representative sections were stained with antibodies targeting CD31 (1:200; Cell Signaling Technology), alpha smooth muscle actin (Acta2) (1:100, Sigma-Aldrich), Ki67 (1:100; Cell Signaling Technology), cleaved caspase-3 (1:200; Cell Signaling Technology), vimentin (1:100; Cell Signaling Technology), and E-cadherin (1:100; Cell Signaling Technology) according to standard protocols (Polasek et al., 2012). Images were captured with a Nikon Eclipse microscope equipped with an Insight CMOS 5.1 digital camera. All image analysis was performed using ImageJ.

### Platinum biodistribution

For the evaluation of relative platinum uptake in various tissues, mice were co-injected via the tail vein with equimolar concentrations of FOLFIRINOX (see dosing schedule in the ‘FOLFIRINOX chemotherapy treatment’ section), followed by animal sacrifice 2 h postinjection using a cardiac terminal blood withdrawal. Blood and tumor were isolated, weighed and acid digested. Platinum levels were measured by high-performance liquid chromatography/ICP-MS, and expressed as amount per wet weight of tissue (Farrar et al., 2017).

### Statistical analysis

Results are expressed as mean±s.d. unless otherwise noted. One-way ANOVA followed by post hoc Tukey tests with two-tailed distribution were performed to analyze data among groups of three or more. Student's *t-*test was used to compare data between the control and one experimental group. *P*<0.05 was considered significant. All calculations were made using GraphPad Prism software.

## Supplementary Material

Supplementary information

## References

[DMM034793C1] Aguirre A. J., Bardeesy N., Sinha M., Lopez L., Tuveson D. A., Horner J., Redston M. S. and DePinho R. A. (2003). Activated Kras and Ink4a/Arf deficiency cooperate to produce metastatic pancreatic ductal adenocarcinoma. *Genes Dev.* 17, 3112-3126. 10.1101/gad.1158703 14681207 PMC305262

[DMM034793C2] Andre F., Schartz N. E. C., Movassagh M., Flament C., Pautier P., Morice P., Pomel C., Lhomme C., Escudier B., Le Chevalier T. et al. (2002). Malignant effusions and immunogenic tumour-derived exosomes. *Lancet* 360, 295-305. 10.1016/S0140-6736(02)09552-1 12147373

[DMM034793C3] Armstrong T., Packham G., Murphy L. B., Bateman A. C., Conti J. A., Fine D. R., Johnson C. D., Benyon R. C. and Iredale J. P. (2004). Type I collagen promotes the malignant phenotype of pancreatic ductal adenocarcinoma. *Clin. Cancer Res.* 10, 7427-7437. 10.1158/1078-0432.CCR-03-0825 15534120

[DMM034793C4] Bachmann J., Heiligensetzer M., Krakowski-Roosen H., Büchler M. W., Friess H. and Martignoni M. E. (2008). Cachexia worsens prognosis in patients with resectable pancreatic cancer. *J. Gastrointest. Surg.* 12, 1193-1201. 10.1007/s11605-008-0505-z 18347879

[DMM034793C5] Ben-David U., Ha G., Tseng Y.-Y., Greenwald N. F., Oh C., Shih J., Mcfarland J. M., Wong B., Boehm J. S., Beroukhim R. et al. (2017). Patient-derived xenografts undergo mouse-specific tumor evolution. *Nat. Genet.* 49, 1567-1575. 10.1038/ng.3967 28991255 PMC5659952

[DMM034793C6] Bramhall S. R., Schulz J., Nemunaitis J., Brown P. D., Baillet M. and Buckels J. A. C. (2002). A double-blind placebo-controlled, randomised study comparing gemcitabine and marimastat with gemcitabine and placebo as first line therapy in patients with advanced pancreatic cancer. *Br. J. Cancer* 87, 161-167. 10.1038/sj.bjc.6600446 12107836 PMC2376102

[DMM034793C7] Chauhan V. P., Martin J. D., Liu H., Lacorre D. A., Jain S. R., Kozin S. V., Stylianopoulos T., Mousa A. S., Han X., Adstamongkonkul P. et al.(2013). Angiotensin inhibition enhances drug delivery and potentiates chemotherapy by decompressing tumour blood vessels. *Nat. Commun.* 4, 2516 10.1038/ncomms3516 24084631 PMC3806395

[DMM034793C8] Erkan M., Michalski C. W., Rieder S., Reiser-Erkan C., Abiatari I., Kolb A., Giese N. A., Esposito I., Friess H. and Kleeff J. (2008). The activated stroma index is a novel and independent prognostic marker in pancreatic ductal adenocarcinoma. *Clin. Gastroenterol. Hepatol.* 6, 1155-1161. 10.1016/j.cgh.2008.05.006 18639493

[DMM034793C9] Farrar C. T., Gale E. M., Kennan R., Ramsay I., Masia R., Arora G., Looby K., Wei L., Kalpathy-Cramer J., Bunzel M. M. et al. (2017). CM-101: type I collagen-targeted MR imaging probe for detection of liver fibrosis. *Radiology* 287, 581-589. 10.1148/radiol.2017170595 29156148 PMC5929363

[DMM034793C10] Feig C., Gopinathan A., Neesse A., Chan D. S., Cook N. and Tuveson D. A. (2012). The pancreas cancer microenvironment. *Clin. Cancer Res.* 18, 4266-4276. 10.1158/1078-0432.CCR-11-3114 22896693 PMC3442232

[DMM034793C11] Fuchs B. C., Wang H., Yang Y., Wei L., Polasek M., Schühle D. T., Lauwers G. Y., Parkar A., Sinskey A. J., Tanabe K. K. et al. (2013). Molecular MRI of collagen to diagnose and stage liver fibrosis. *J. Hepatol.* 59, 992-998. 10.1016/j.jhep.2013.06.026 23838178 PMC3805694

[DMM034793C12] Heinemann V., Haas M. and Boeck S. (2013). Neoadjuvant treatment of borderline resectable and non-resectable pancreatic cancer. *Ann. Oncol.* 24, 2484-2492. 10.1093/annonc/mdt239 23852311

[DMM034793C13] Hingorani S. R., Wang L., Multani A. S., Combs C., Deramaudt T. B., Hruban R. H., Rustgi A. K., Chang S. and Tuveson D. A. (2005). Trp53R172H and KrasG12D cooperate to promote chromosomal instability and widely metastatic pancreatic ductal adenocarcinoma in mice. *Cancer Cell* 7, 469-483. 10.1016/j.ccr.2005.04.023 15894267

[DMM034793C14] Hoover M., Adamian Y., Brown M., Maawy A., Chang A., Lee J., Gharibi A., Katz M. H., Fleming J., Hoffman R. M. et al. (2017). A novel method for RNA extraction from FFPE samples reveals significant differences in biomarker expression between orthotopic and subcutaneous pancreatic cancer patient-derived xenografts. *Oncotarget* 8, 5885-5894. 10.18632/oncotarget.11809 27602776 PMC5351598

[DMM034793C15] Ijichi H., Chytil A., Gorska A. E., Aakre M. E., Fujitani Y., Fujitani S., Wright C. V. E. and Moses H. L. (2006). Aggressive pancreatic ductal adenocarcinoma in mice caused by pancreas-specific blockade of transforming growth factor-beta signaling in cooperation with active Kras expression. *Genes Dev.* 20, 3147-3160. 10.1101/gad.1475506 17114585 PMC1635149

[DMM034793C16] Indolfi L., Ligorio M., Ting D. T., Xega K., Tzafriri A. R., Bersani F., Aceto N., Thapar V., Fuchs B. C., Deshpande V. et al. (2016). A tunable delivery platform to provide local chemotherapy for pancreatic ductal adenocarcinoma. *Biomaterials* 93, 71-82. 10.1016/j.biomaterials.2016.03.044 27082874 PMC4849535

[DMM034793C17] Ireland L., Santos A., Ahmed M. S., Rainer C., Nielsen S. R., Quaranta V., Weyer-Czernilofsky U., Engle D. D., Perez-Mancera P. A., Coupland S. E. et al. (2016). Chemoresistance in pancreatic cancer is driven by stroma-derived insulin-like growth factors. *Cancer Res.* 76, 6851-6863. 10.1158/0008-5472.CAN-16-1201 27742686 PMC5321488

[DMM034793C18] Kim M. P., Evans D. B., Wang H., Abbruzzese J. L., Fleming J. B. and Gallick G. E. (2009). Generation of orthotopic and heterotopic human pancreatic cancer xenografts in immunodeficient mice. *Nat. Protoc.* 4, 1670-1680. 10.1038/nprot.2009.171 19876027 PMC4203372

[DMM034793C19] Knudsen E. S., Balaji U., Mannakee B., Vail P., Eslinger C., Moxom C., Mansour J. and Witkiewicz A. K. (2017). Pancreatic cancer cell lines as patient-derived avatars: genetic characterisation and functional utility. *Gut* 67, 508-520. 10.1136/gutjnl-2016-313133 28073890 PMC5868284

[DMM034793C20] Koay E. J., Baio F. E., Ondari A., Truty Mark J., Cristini V., Thomas R. M., Chen R., Chatterjee D., Kang Y., Zhang J. et al. (2014). Intra-tumoral heterogeneity of gemcitabine delivery and mass transport in human pancreatic cancer. *Phys. Biol.* 11, 065002 10.1088/1478-3975/11/6/065002 25427073 PMC4266401

[DMM034793C21] Kohler H. and Lankisch P. G. (1987). Acute pancreatitis and hyperamylasaemia in pancreatic carcinoma. *Pancreas* 2, 117-119. 10.1097/00006676-198701000-00018 2437571

[DMM034793C22] Kojima K., Vickers S. M., Adsay N. V., Jhala N. C., Kim H.-G., Schoeb T. R., Grizzle W. E. and Klug C. A. (2007). Inactivation of Smad4 accelerates Kras(G12D)-mediated pancreatic neoplasia. *Cancer Res.* 67, 8121-8130. 10.1158/0008-5472.CAN-06-4167 17804724

[DMM034793C23] Lim K.-H., Chung E., Khan A., Cao D., Linehan D., Ben-Josef E. and Wang-Gillam A. (2012). Neoadjuvant therapy of pancreatic cancer: the emerging paradigm? *Oncologist* 17, 192-200. 10.1634/theoncologist.2011-0268 22250057 PMC3286168

[DMM034793C24] Lin A. and Feller E. R. (1990). Pancreatic carcinoma as a cause of unexplained pancreatitis: report of ten cases. *Ann. Intern. Med.* 113, 166-167. 10.7326/0003-4819-113-2-166 1694415

[DMM034793C25] Linder S., Castanos-Velez E., Von Rosen A. and Biberfeld P. (2001). Immunohistochemical expression of extracellular matrix proteins and adhesion molecules in pancreatic carcinoma. *Hepato-Gastroenterology* 48, 1321-1327. 11677955

[DMM034793C26] Michaelis K. A., Zhu X., Burfeind K. G., Krasnow S. M., Levasseur P. R., Morgan T. K. and Marks D. L. (2017). Establishment and characterization of a novel murine model of pancreatic cancer cachexia. *J. Cachexia Sarcopenia Muscle* 8, 824-838. 10.1002/jcsm.12225 28730707 PMC5659050

[DMM034793C27] Mohelnikova-Duchonova B., Brynychova V., Oliverius M., Honsova E., Kala Z., Muckova K. and Soucek P. (2013). Differences in transcript levels of ABC transporters between pancreatic adenocarcinoma and nonneoplastic tissues. *Pancreas* 42, 707-716. 10.1097/MPA.0b013e318279b861 23462326

[DMM034793C28] Mollenhauer J., Roether I. and Kern H. F. (1987). Distribution of extracellular matrix proteins in pancreatic ductal adenocarcinoma and its influence on tumor cell proliferation in vitro. *Pancreas* 2, 14-24. 10.1097/00006676-198701000-00003 3554225

[DMM034793C29] Moore M. J., Hamm J., Dancey J., Eisenberg P. D., Dagenais M., Fields A., Hagan K., Greenberg B., Colwell B., Zee B. et al. (2003). Comparison of gemcitabine versus the matrix metalloproteinase inhibitor BAY 12-9566 in patients with advanced or metastatic adenocarcinoma of the pancreas: a phase III trial of the national cancer institute of Canada clinical trials group. *J. Clin. Oncol.* 21, 3296-3302. 10.1200/JCO.2003.02.098 12947065

[DMM034793C30] Mujica V. R., Barkin J. S. and Go V. L. (2000). Acute pancreatitis secondary to pancreatic carcinoma. Study group participants. *Pancreas* 21, 329-332. 10.1097/00006676-200011000-00001 11075985

[DMM034793C31] Nakamura T., Fidler I. J. and Coombes K. R. (2007). Gene expression profile of metastatic human pancreatic cancer cells depends on the organ microenvironment. *Cancer Res.* 67, 139-148. 10.1158/0008-5472.CAN-06-2563 17210693

[DMM034793C32] Neesse A., Frese K. K., Bapiro T. E., Nakagawa T., Sternlicht M. D., Seeley T. W., Pilarsky C., Jodrell D. I., Spong S. M. and Tuveson D. A. (2013). CTGF antagonism with mAb FG-3019 enhances chemotherapy response without increasing drug delivery in murine ductal pancreas cancer. *Proc. Natl. Acad. Sci. USA* 110, 12325-12330. 10.1073/pnas.1300415110 23836645 PMC3725120

[DMM034793C33] Olive K. P., Jacobetz M. A., Davidson C. J., Gopinathan A., Mcintyre D., Honess D., Madhu B., Goldgraben M. A., Caldwell M. E., Allard D. et al. (2009). Inhibition of Hedgehog signaling enhances delivery of chemotherapy in a mouse model of pancreatic cancer. *Science* 324, 1457-1461. 10.1126/science.1171362 19460966 PMC2998180

[DMM034793C34] Ozdemir B. C., Pentcheva-Hoang T., Carstens J. L., Zheng X., Wu C.-C., Simpson T. R., Laklai H., Sugimoto H., Kahlert C., Novitskiy S. V. et al. (2015). Depletion of carcinoma-associated fibroblasts and fibrosis induces immunosuppression and accelerates pancreas cancer with reduced survival. *Cancer Cell* 28, 831-833. 10.1016/j.ccell.2015.11.002 28843279

[DMM034793C35] Polasek M., Fuchs B. C., Uppal R., Schühle D. T., Alford J. K., Loving G. S., Yamada S., Wei L., Lauwers G. Y., Guimaraes A. R. et al. (2012). Molecular MR imaging of liver fibrosis: a feasibility study using rat and mouse models. *J. Hepatol.* 57, 549-555. 10.1016/j.jhep.2012.04.035 22634342 PMC3423553

[DMM034793C36] Rubio-Viqueira B., Jimeno A., Cusatis G., Zhang X., Iacobuzio-Donahue C., Karikari C., Shi C., Danenberg K., Danenberg P. V., Kuramochi H. et al. (2006). An in vivo platform for translational drug development in pancreatic cancer. *Clin. Cancer Res.* 12, 4652-4661. 10.1158/1078-0432.CCR-06-0113 16899615

[DMM034793C37] Ryan D. P., Hong T. S. and Bardeesy N. (2014). Pancreatic adenocarcinoma. *N Engl. J. Med.* 371, 1039-1049. 10.1056/NEJMra1404198 25207767

[DMM034793C38] SEER Stat Fact Sheets (2014). Pancreas Cancer. Surveillance, Epidemiology, and End Results Program (SEER): National Cancer Institute.

[DMM034793C39] Son J., Lyssiotis C. A., Ying H., Wang X., Hua S., Ligorio M., Perera R. M., Ferrone C. R., Mullarky E., Shyh-Chang N. et al. (2013). Glutamine supports pancreatic cancer growth through a KRAS-regulated metabolic pathway. *Nature* 496, 101-105. 10.1038/nature12040 23535601 PMC3656466

[DMM034793C40] Stromnes I. M., Delgiorno K. E., Greenberg P. D. and Hingorani S. R. (2014). Stromal reengineering to treat pancreas cancer. *Carcinogenesis* 35, 1451-1460. 10.1093/carcin/bgu115 24908682 PMC4076816

[DMM034793C41] Thompson C. B., Shepard H. M., O'connor P. M., Kadhim S., Jiang P., Osgood R. J., Bookbinder L. H., Li X., Sugarman B. J., Connor R. J. et al. (2010). Enzymatic depletion of tumor hyaluronan induces antitumor responses in preclinical animal models. *Mol. Cancer Ther.* 9, 3052-3064. 10.1158/1535-7163.MCT-10-0470 20978165

[DMM034793C42] Tinder T. L., Subramani D. B., Basu G. D., Bradley J. M., Schettini J., Million A., Skaar T. and Mukherjee P. (2008). MUC1 enhances tumor progression and contributes toward immunosuppression in a mouse model of spontaneous pancreatic adenocarcinoma. *J. Immunol.* 181, 3116-3125. 10.4049/jimmunol.181.5.3116 18713982 PMC2625292

[DMM034793C43] Tseng W. W., Winer D., Kenkel J. A., Choi O., Shain A. H., Pollack J. R., French R., Lowy A. M. and Engleman E. G. (2010). Development of an orthotopic model of invasive pancreatic cancer in an immunocompetent murine host. *Clin. Cancer Res.* 16, 3684-3695. 10.1158/1078-0432.CCR-09-2384 20534740 PMC3085509

[DMM034793C44] Tuveson D. A., Zhu L., Gopinathan A., Willis N. A., Kachatrian L., Grochow R., Pin C. L., Mitin N. Y., Taparowsky E. J., Gimotty P. A. et al. (2006). Mist1-KrasG12D knock-in mice develop mixed differentiation metastatic exocrine pancreatic carcinoma and hepatocellular carcinoma. *Cancer Res.* 66, 242-247. 10.1158/0008-5472.CAN-05-2305 16397237

[DMM034793C45] Zhan B., Wen S., Lu J., Shen G., Lin X., Feng J. and Huang H. (2017). Identification and causes of metabonomic difference between orthotopic and subcutaneous xenograft of pancreatic cancer. *Oncotarget* 8, 61264-61281. 28977862 10.18632/oncotarget.18057 PMC5617422

