# Supplementary material for: Orthotopic and heterotopic murine models of pancreatic cancer and their different responses to FOLFIRINOX chemotherapy
Source: Dis Model Mech. 2018 Jul 30;11(7):dmm034793. doi: 10.1242/dmm.034793 (PMC6078400; doi:10.1242/dmm.034793)
Supplement: Supplementary information [file dmm-11-034793-s1.pdf]

FIRST PERSON

First person – Derek Erstad

First Person is a series of interviews with the first authors of a selection of papers published in Disease Models & Mechanisms, helping early-career researchers promote themselves alongside their papers. Derek Erstad is first author on 'Orthotopic and heterotopic murine models of pancreatic cancer and their different responses to FOLFIRINOX chemotherapy', published in DMM. Derek is a Surgical Resident and Clinical Research Fellow under the supervision of Kenneth K. Tanabe and Bryan C. Fuchs at Massachusetts General Hospital, Boston, USA, investigating the use of novel chemotherapeutic agents in combination with surgical resection for solid gastrointestinal malignancies.

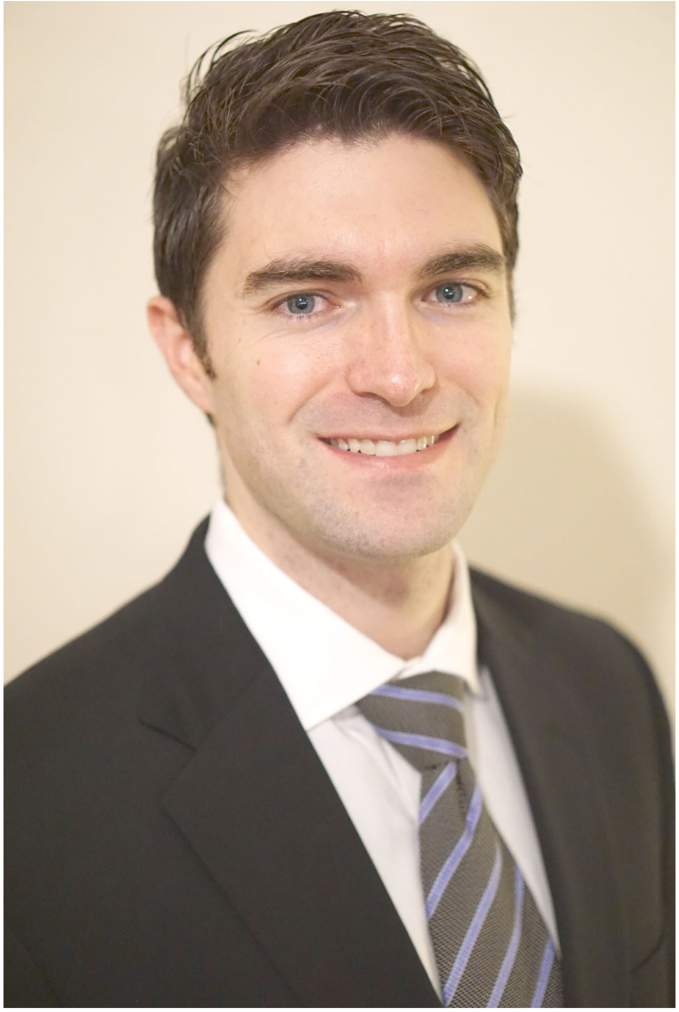

Derek Erstad

How would you explain the main findings of your paper to non-scientific family and friends?

Our research team is interested in studying pancreatic cancer, which is the fourth leading cause of cancer-related death in the United States. One way to study pancreatic cancer is by developing models that recreate the disease process in animals like mice. Although animal models are imperfect, they allow us to better understand how cancers behave and whether investigational new therapies might be of value in humans. For pancreatic cancer, there are multiple models that have been created in mice. In one model, called an orthotopic model, pancreatic cancer cells are surgically implanted directly into the pancreas; in contrast, in a heterotopic model, pancreatic cancer cells are placed into the soft tissue of the flank. However, because mouse tissues are small, implanting cancer cells into specific organs can be challenging, and cells may leak from the injection site, ruining the model. Given this context, one goal of our study was to determine the optimal surgical technique for creating orthotopic and heterotopic pancreatic tumors in mice that minimize experimental error. We compared commonly used surgical techniques and identified a series of maneuvers that yielded the greatest success. Based on these observations, we were able to provide highly detailed analyses of surgical technique, which is currently lacking in the field.

> **"The key takeaway is that the location of tumor implantation in a mouse indeed matters and should be acknowledged in the design of any given experiment."**

A second goal of this study was to determine whether there are significant differences in the appearance and behavior of pancreatic tumors based on their location of implantation. We found that tumors implanted in the pancreas grew faster and larger compared to tumors implanted in the flank. In contrast, flank tumors had higher levels of scar tissue, which we call fibrosis, within the tumor. The amount of fibrosis in pancreatic cancer has been associated with responsiveness to therapy and survival in humans, so this latter point is particularly relevant to model design. Finally, we observed that flank tumors are less responsive to chemotherapy than tumors located in the pancreas, which might be related to the differences in the amount of fibrosis present. The key takeaway is that the location of tumor implantation in a mouse indeed matters and should be acknowledged in the design of any given experiment.

Derek Erstad's contact details: Massachusetts General Hospital, Department of Surgery, 55 Fruit Street, Boston, MA 02114, USA.
E-mail: derstad@partners.org

What are the potential implications of these results for your field of research?

Many pancreatic cancer researchers abandon the orthotopic model system because they cannot overcome the technical failure of leakage, which results in carcinomatosis. Although other groups have published on general methodologic principles for orthotopic tumor implantation, our study addresses the particular issue of leakage and methods for troubleshooting it. Therefore, we hope that our findings will help researchers struggling with this model to develop a reliably working system.

The second set of implications from our study regards the biologic characterization of tumors based on implantation location. Flank tumors are very commonly used for pancreatic cancer research because they are technically easier to establish than pancreatic implantation, which requires an abdominal surgery. Flank tumors can

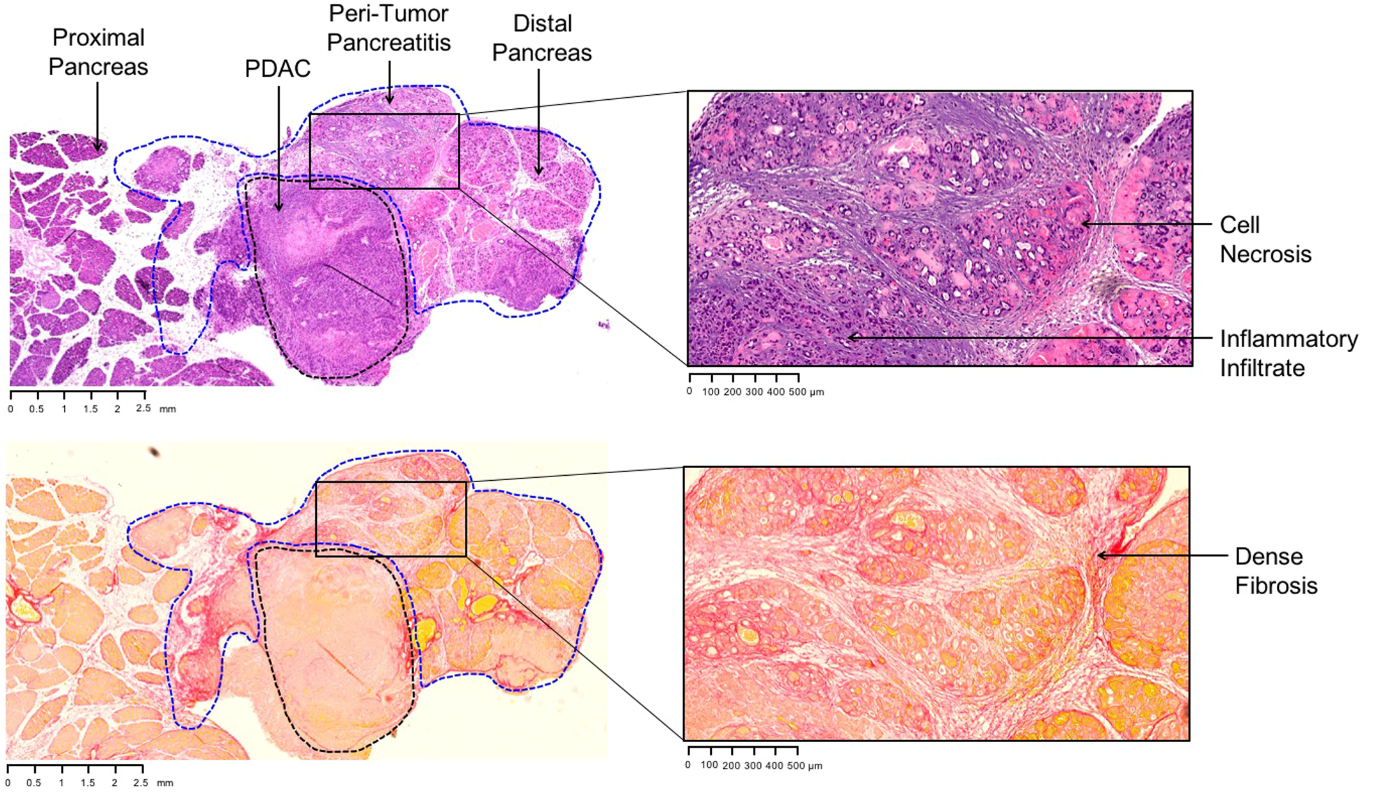

Mouse pancreas with a syngeneic, orthotopic tumor implantation after two weeks of growth. Hematoxylin and Eosin staining is shown in the top image, and Sirius Red collagen staining is shown in the bottom image. A unique advantage of the orthotopic pancreatic cancer model, as opposed to the commonly used flank tumor technique, is the recapitulation of peri-tumor pancreatitis, which is a common histologic feature of human cancers. A second unique feature of pancreatic tumors is their intense desmoplastic reaction, evidenced by the high degree of collagen deposition in the tumor microenvironment.

also be easily observed as a bulge under the skin and can be measured after therapy with calipers, which are additional advantages. However, there has been minimal research into the biological differences between flank and orthotopic tumors, and how those differences might influence experimental outcomes. We observed that flank tumors are more desmoplastic and chemoresistant to FOLFIRINOX, a regimen commonly given to human patients, compared to orthotopic tumors. These phenotypic differences can be tailored to a particular experimental question by researchers, but they also provide evidence to support further investigation into microenvironmental differences between flank and orthotopic locations that contribute to desmoplasia and chemoresistance.

What are the main advantages and drawbacks of the model system you have used as it relates to the disease you are investigating?

The various advantages of the syngeneic, orthotopic murine PDAC model have been discussed above; however, there are two main drawbacks. The first is that it is not a model of carcinogenesis like a genetically engineered mouse model, e.g. KPC, in which tumors spontaneously generate in the pancreas. The second limitation is that the source of cancer cells is generally from a cell line, which can appear genetically distinct from adenocarcinoma cells isolated from a primary tumor.

What has surprised you the most while conducting your research?

The biggest surprise was that tumor location impacts sensitivity to FOLFIRINOX chemotherapy. Because flank tumors are inherently more desmoplastic and avascular, we initially suspected this might be a consequence of reduced drug penetration. However, when we tested for chemotherapy uptake into tumor tissue using mass spectrophotometry, we did not observe a difference between heterotopic and orthotopic locations. Given that the same cell line was injected into both locations, these observations indicate that differences in respective tumor microenvironments impact susceptibility to cytotoxic chemicals.

Describe what you think is the most significant challenge impacting your research at this time and how will this be addressed over the next 10 years?

In the field of pancreatic cancer research, I think one critical challenge is that we need a better understanding of early micrometastatic dissemination. For example, a patient with early stage pancreatic cancer can undergo a complete resection with negative margins and subsequently have no evidence of residual disease by all available metrics. However, the statistical probability that this person still dies of disease within the next five years is very high. This means that by the time a patient clinically presents with disease, even if caught early, they are likely to already have microscopic metastases, which are key drivers of mortality. We know that certain clinicopathological traits of the primary tumor predict outcome, which means that there is likely some degree of biologic overlap between the primary and metastatic spread. However, as basic scientists, focusing mainly on the biology and treatment-related changes of the primary lesion might be missing the mark in terms of translational impact. I think this problem will be addressed in the coming years through a combination of

technologies. For example, methods for isolating individual tumor cells and single-cell nucleic acid sequencing technology might provide a more direct path to understanding the nature of metastasis, and as a result, more promising therapeutic targets.

What changes do you think could improve the professional lives of early-career scientists?

"As a scientific community, the onus is on us to share and make resources open source when possible."

I think the main challenge with early-career scientists is funding. In the United States, government funding through the National Institutes of Health (NIH) is increasingly competitive. Simultaneously, research is increasingly expensive as more sophisticated technologies have become experimental standards, and as a result, the financial constraints placed on young investigators is very high. A major consequence of financial constraint is loss of time. As the percentage of funded applications continues to decrease for most grant programs, young investigators are forced to write for an increasing number of grants, which is a time-consuming process that assuredly impacts livelihood. I am a physician by training, so my perspective is from that of a clinician-scientist, and it appears that maintaining a scientific career under these pressures has become increasingly difficult. Unfortunately, I am not sure significant change can or will realistically occur in the near future. At a policy level, funding initiatives dedicated specifically to early investigators are an obvious and important means of addressing this problem. As a scientific community, the onus is on us to share and make resources open source when possible. These changes might help to reduce at least some of the stress faced by early-career scientists.

What's next for you?

I am finishing my surgical training at the Massachusetts General Hospital in Boston and will be applying for surgical oncology fellowship over the next year.

Reference

Erstad, D. J., Sojoodi, M., Taylor, M. S., Ghoshal, S., Razavi, A. A., Graham-O'Regan, K. A., Bardeesy, N., Ferrone, C. R., Lanuti, M., Caravan P., Tanabe, K. K. and Fuchs, B. C. (2018). Orthotopic and heterotopic murine models of pancreatic cancer and their different responses to FOLFIRINOX chemotherapy. Dis. Model. Mech. **11**: dmm034793.
